# Development of metabolic signatures of plant-rich dietary patterns using plant-derived metabolites

**DOI:** 10.1007/s00394-024-03511-x

**Published:** 2024-11-28

**Authors:** Yong Li, Yifan Xu, Melanie Le Sayec, Tim D. Spector, Claire J. Steves, Cristina Menni, Rachel Gibson, Ana Rodriguez-Mateos

**Affiliations:** 1 Department of Nutritional Sciences, School of Life Course and Population Sciences, Faculty of Life Sciences and Medicine, King’s College London, London, WC2R 2LS UK; 2 Department of Twin Research and Genetic Epidemiology, School of Life Course and Population Sciences, Faculty of Life Sciences and Medicine, King’s College London, London, WC2R 2LS UK; 3 Department of Pathophysiology and Transplantation, Università Degli Studi di Milano, Milan, 20122 Italy

**Keywords:** Plant-rich dietary pattern, Metabolic signature, Dietary assessment, Metabolomics

## Abstract

**Background:**

Diet is an important modifiable lifestyle factor for human health, and plant-rich dietary patterns are associated with lower risk of non-communicable diseases in numerous studies. However, objective assessment of plant-rich dietary exposure in nutritional epidemiology remains challenging.

**Objectives:**

This study aimed to develop and evaluate metabolic signatures of the most widely used plant-rich dietary patterns using a targeted metabolomics method comprising 108 plant food metabolites.

**Methods:**

A total of 218 healthy participants were included, aged 51.5 ± 17.7 years, with 24 h urine samples measured using ultra-high-performance liquid chromatography–mass spectrometry. The validation dataset employed three sample types to test the robustness of the signature, including 24 h urine (*n* = 88), plasma (*n* = 195), and spot urine (*n* = 198). Adherence to the plant-rich diet was assessed using a priori plant-rich dietary patterns calculated using Food Frequency Questionnaires. A combination of metabolites evaluating the adherence to a specific diet was identified as metabolic signature. We applied linear regression analysis to select the metabolites significantly associated with dietary patterns (adjusting energy intake), and ridge regression to estimate penalized weights of each candidate metabolite. The correlation between metabolic signature and the dietary pattern was assessed by Spearman analysis (FDR < 0.05).

**Results:**

The metabolic signatures consisting of 42, 22, 35, 15, 33, and 33 predictive metabolites across different subclasses were found to be associated with adherence to Amended Mediterranean Score (A-MED), Original MED (O-MED), Dietary Approaches to Stop Hypertension (DASH), Mediterranean-DASH Intervention for Neurodegenerative Delay (MIND), healthy Plant-based Diet Index (hPDI) and unhealthy PDI (uDPI), respectively. The overlapping and distinct predictive metabolites across six dietary patterns predominantly consisted of phenolic acids (*n* = 38), including 14 cinnamic acids, 14 hydroxybenzoic acids, seven phenylacetic acids, and three hippuric acids. Six metabolites were included in all signatures, including two lignans: enterolactone-glucuronide, enterolactone-sulfate, and four phenolic acids: cinnamic acid, cinnamic acid-4’-sulfate, 2’-hydroxycinnamic acid, and 4-methoxybenzoic acid-3-sulfate. The established signatures were robustly correlated with dietary patterns in the validation datasets (*r* = 0.13–0.40, FDR < 0.05).

**Conclusions:**

We developed and evaluated a set of metabolic signatures that reflected the adherence to plant-rich dietary patterns, suggesting the potential of these signatures to serve as an objective assessment of free-living eating habits.

**Supplementary Information:**

The online version contains supplementary material available at 10.1007/s00394-024-03511-x.

## Introduction

Plant-based diets are typically characterised by high consumption of plant foods, including fruits, vegetables, wholegrains, nuts, legumes, and vegetable oils [1, 2]. Studies suggest that common plant-rich dietary patterns are associated with preventing non-communicable diseases, especially cardiometabolic diseases [3]. A number of plant-based diet indices have been developed to reflect the adherence to certain dietary patterns and evaluate diet quality regarding health outcomes, such as the *Mediterranean Diet score* (MDS) [4], *Dietary Approaches to Stop Hypertension* (DASH) [5], *Plant-based diet index* (PDI) [6], and *The Mediterranean-DASH Diet Intervention for Neurodegenerative Delay* (MIND) [7, 8]. However, a disadvantage of this approach is that it relies on the use of dietary assessment methods including food frequency questionnaires (FFQs), food diaries, or dietary recalls. These methods rely on self-report assessment, which is subject to bias [9]. Due to this important limitation, extensive efforts have been made to develop more objective dietary assessment techniques, such as validated biological biomarkers of food intake as a more objective assessment method to monitor dietary exposure [10].

Only a limited number of food biomarkers have been proposed using metabolomics approaches in epidemiological studies, through the analysis of associations between biomarkers and dietary intake exposure [11]. These biomarkers include food-derived metabolites and catabolites measured in plasma, serum or urine using analytical techniques such as Liquid Chromatography-Mass Spectrometry (LC-MS) or Nuclear Magnetic Resonance (NMR). To date, a few biomarkers, such as proline betaine, naringenin, and hesperetin, have been proposed as biomarkers of specific fruit and vegetable intake, and α-carotene, β-carotene, and chlorogenic acid as biomarkers of total fruit and vegetable intake in nutrition epidemiological studies [11, 12]. In addition to a more objective assessment that does not rely on self-reported intake, metabolomic profiling also reflects the existing inter-individual variability in metabolism and may be a better indicator of overall dietary exposure. Thus, metabolomics through high-throughput profiling holds the potential to capture adherence to a predefined dietary pattern.

Based on metabolomic profiling, the use of metabolic signatures (i.e., a combination of metabolites that evaluates the adherence to a specific diet [13]) for characterising dietary patterns has recently been proposed [14]. Supervised machine learning methods, such as elastic net regression, identified a plasma metabolic signature consisting of 67 metabolites that correlated with adherence to the Mediterranean diet in two cohorts from Spain and the US [13]. A metabolic signature consisting of 66 metabolites has also been proposed to reflect adherence to the Mediterranean diet in a cohort recruited in the UK [15]. The two signatures proposed overlapped in several metabolites, including proline, threonine, carnitine, creatinine, citrulline, and glutamate. In a systematic review summarizing the use of metabolomics for dietary intake, only 16 studies evaluated the metabolite signature in relation to dietary patterns, including vegetarian and lactovegetarian diets, omnivorous diets, western dietary patterns, prudent dietary patterns, Nordic diet, and Mediterranean diet [14]. There is still a lack of systematic exploration of metabolic signatures of plant-based dietary patterns.

Here we investigated and developed metabolic signatures for the most widely used plant-based dietary patterns, measured by a validated ultra-High-Performance LC-MS (UHPLC-MS) targeted method for plant food metabolites in urine samples and FFQs in 2 UK based cohorts.

## Methods

### Study population

The baseline data from 9 clinical studies conducted at King’s College London between 2017 and 2021 were combined in the POLYINTAKE cohort (Ethics number, RESCM-17/18-5283; HR-15/16-3739; HR-17/18-5338; HR-18/19-9091; HR-18/19-8999; HR-17/18-5703; RESCM-18/19-9036; HR-17/18-5353; HR-19/20-14771; Trial registration number, NCT03434574; NCT03041961; NCT03592966; NCT04084457; NCT04179136; NCT03553225; NCT03995602; NCT03573414; NCT04276974). These data were combined due to standard baseline data collection methods across all participants. The studies were conducted according to the Declaration of Helsinki, and all participants provided informed written consent, and all consented to data used in future research studies.

Participants that completed an FFQ and provided plasma and/or urine samples were included in this cross-sectional study. To eliminate the outliers related to dietary intake, some of the participants were excluded from the analysis based on the following reasons as previously described [16]: (i) more than ten missing food items from the FFQ; (ii) Females with fewer than 500 kcal/d and higher than 3,500 kcal/d and males with fewer than an average of 800 kcal/d and higher than 4,000 kcal/d [17]; (iii) The ratio of energy intake (EI): basal metabolic rate (BMR, estimated by the Harris-Benedict equation) out of mean ± 2 standard deviations (SD) of this population according to the Goldberg method [18]. A total of 218 participants with FFQ and 24 h urine samples were included, of which 195 had additional plasma samples collected and analyzed.

Participants from the sub-cohort of an independent Aronia Berry Consumption on Blood Pressure study (ABP) were used as an internal validation set, which included 88 participants after exclusions. The trial was approved by King’s College London’s Ethics Committee (RESCM-21/22–26721) with the trial registered at ClinicalTrials.gov NCT03434574. Plasma samples of the POLYNTAKE cohort were also used as the internal validation set (*n* = 195). TwinsUK cohort with 198 participants after exclusion was used as an external validation set with the ethical approval from the NHS Research Ethics Committee at the Department of Twin Research and Genetic Epidemiology, King’s College London (the Healthy Ageing Twin Study (H.A.T.S) 07/H0802/84) and the NRES Committee London-Westminster (Flora Twin Study reference 12/LO/0227) [19].

### Dietary intake assessment and dietary patterns generation

Each participant in the POLYNTAKE cohort completed the European Prospective Investigation into Diet and Cancer (EPIC) Norfolk FFQ validated against key nutrient intakes [20]. FFQ EPIC and Nutrition Tool for Analysis (FETA) software, which involved composition data of 290 foods from the UK food composition database McCance and Widdowson’s ‘The Composition of Foods’ (5th edition) and its associated supplements [21] were used in this research to estimate energy intake. Five commonly used plant-rich dietary patterns were chosen to evaluate the overall food constitution based on the predefined dietary scoring formula: DASH [5], PDI (along with healthy PDI (hPDI) and unhealthy PDI (uPDI)) [6], *Original Mediterranean Score* (O-MED) [4], *Amended Mediterranean Score* (A-MED) [22], and MIND [8]. The detailed food group descriptions and the calculation method for each dietary score were previously reported [16]. These dietary patterns share a common emphasis on plant-based foods, with fruits and vegetables serving as fundamental components. However, specific distinctions are present, such as the differentiation between green leafy and other vegetables and only berries in the fruit category for the MIND. This specificity is due to evidence indicating the cognitive benefits of green leafy vegetables and the protective effects of berries against cognitive decline rather than overall fruit consumption [8]. Furthermore, most of these patterns employ a scoring system based on relative food intake, categorizing consumption into quintiles (e.g., DASH [23] and PDI [24]) or medians (e.g., A-MED [22] and O-MED [25]). Participants from the TwinsUK cohort also completed the validated EPIC Norfolk FFQ (available n = 198) [19].

### Sample collection and metabolite analysis

In the POLYNTAKE cohort, the 24 h urine (*n* = 218) and fasting plasma samples (*n* = 195) were collected to analyse plant food metabolite levels. The 24 h urine samples were collected using plastic containers (2 L) stored in a bag with ice packs to keep the urine in a cool condition during and after collection. The participants were instructed to collect urine starting from the second urine of the day before the visit and finishing with the first urine on the day of the study visit. Once received, the total volume of the 24 h urine was measured by a volumetric cylinder. As for plasma sample collection, the participants were instructed to fast for eight hours before coming to the research facility. The fasting blood samples were collected through venepuncture into ethylenediaminetetraacetic acid (EDTA) vacutainers (10 ml, BD) by trained phlebotomists. The urine and blood samples were centrifuged at 1800 g for 15 min at 4 °C right after the collection. Plasma was obtained from the supernatant of the blood samples, and urine and plasma samples were spiked with 0.2% formic acid (Thermo Fisher, LC-MS grade, Loughborough, UK) before storing at -80 °C in labelled plastic tubes until analysis. The detailed processing and analysis of the samples followed a validated method [26]. Samples were thawed on ice for 0.5–1.0 h until fully defrosted and then centrifuged at 15,000 g for 15 min at 4 ℃ using a temperature-controlled microtube centrifuge (5417 R, Eppendorf, Hamburg, Germany). Urine samples were diluted five folds with HPLC water (Sigma-Aldrich, Steinheim, Germany), while plasma samples were processed directly. A total of 108 urinary metabolites and 116 plasma metabolites were identified and quantified using authentic chemical standards. The UHPLC-MS analysis of the samples and standard mixes was achieved with a triple-quadruple mass spectrometer (SHIMADZU 8060, Shimadzu, Kyoto, Japan) coupled with a UHPLC system (Shimadzu, Kyoto, Japan). The samples (5 µL) were injected by autosampler (SIL-30AC, Shimadzu, Kyoto, Japan) through a Raptor Biphenyl column 2.1 × 50 mm, 1.8 μm (Restek, Bellefonte, USA) coupled with a compatible guard cartridge 5 × 2.1 mm, 2.7 μm (Restek, Bellefonte, USA) before reaching a heated ESI source. The MS parameters and multiple reaction monitoring (MRM) method parameters of the target compounds were detailed previously [26]. The peak area ratio of the target compounds to the internal standard taxifolin was used in the quantification to minimise the influence of changes in device performance on the results. The LabSolutions software (SHIMADZU, Kyoto, Japan) was used in the peak integration, and the Microsoft Excel (Excel 2020, Microsoft, USA) was used for concentration calculation.

The spot urine samples in the TwinsUK cohort (*n* = 198) were processed and analysed in the same way as the samples from the POLYNTAKE study as described in previous study [19]. The urinary creatinine levels were measured by Affinity Biomarker Labs (London, UK) using the Jaffe method and the concentrations of the metabolites (nM) were adjusted by the creatinine levels (mg/L) into mmol/g creatinine [19].

### Statistical analysis

The statistical analysis was conducted using R (version 4.1.2) [27]. Dietary scores were normally distributed in the population. The metabolite levels were log-transformed and adjusted for batch effect using the ComBat method [28] with the sva package in R before entering the model. The ComBat method is an empirical Bayes method developed originally for removing batch effect in the microarray data in gene sequencing, and it has then been applied to metabolomics analysis [29]. The energy intake levels estimated from FFQs were adjusted as confounders in the linear regression model to explore the association between plant-rich dietary scores and metabolites with the lm. beta package in R. All analyses were adjusted for multiple testing (Benjamini and Hochberg False Discovery Rate (FDR) < 0.05, FDR suggestive significant 0.05 ~ 0.10) [30]. The metabolites with significant associations were chosen for the next step.

Metabolic signatures for each plant-rich dietary pattern were generated to collectively represent the adherence to the overall dietary patterns based on the selected significant metabolites. The dietary pattern with less than ten metabolites significantly associated was discarded in this step (PDI). Ridge regression was applied to estimate the penalized weights of the candidate metabolites [31]. The metabolic signature of each individual dietary score was constructed based on the weights of all selected metabolites in derivation and validation datasets. The validation datasets included 24 h urine from the internal sub-cohort ABP study, plasma samples from the POLYNTAKE cohort, and external spot urine samples from the TwinsUK external dataset.

**Fig. 1:**
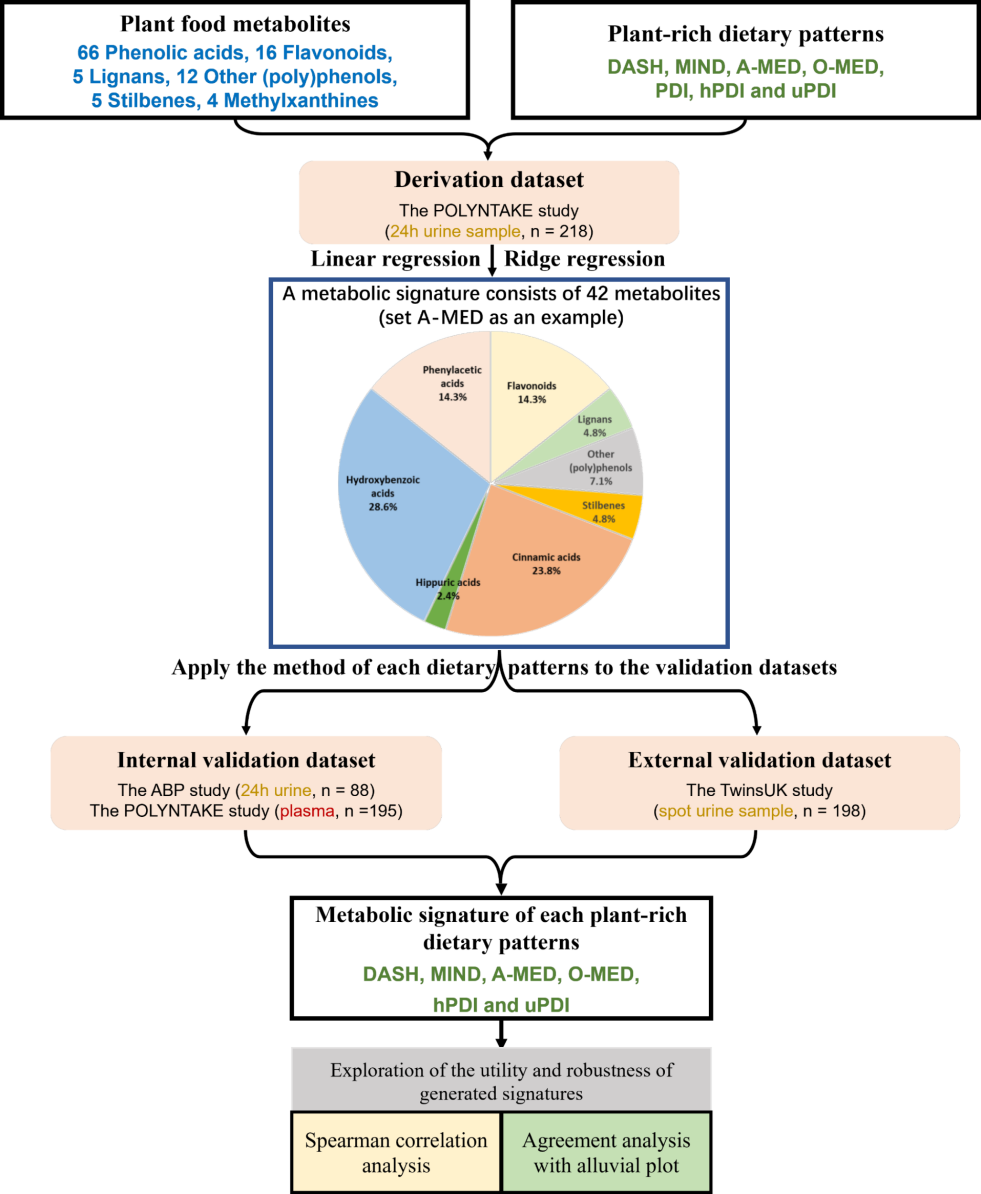
The flowchart of the analysis approach (generation and validation) of the metabolic signature adherence to the plant-rich dietary patterns. DASH, Dietary Approaches to Stop Hypertension; MIND, Mediterranean-DASH Intervention for Neurodegenerative Delay; O-MED, Original Mediterranean Score; A-MED, Amended Mediterranean Score; PDI, Plant-based Diet Index; hPDI, Healthy Plant-based Diet Index; uPDI, unhealthy Plant-based Diet Index

The correlation between plant-rich dietary patterns and their metabolic signature was assessed by Spearman correlation analyses in derivation and validation datasets to explore the utility and robustness of the generated metabolite signatures. To further test the agreement between plant-rich dietary patterns and metabolite signatures, an alluvial plot was constructed to illustrate how the targeted population in both datasets was allocated across categorical dimensions. The detailed flowchart of the analysis is depicted in Fig. 1.

## Results

The demographic, sample, and dietary score characteristics of the participants of the POLYNTAKE cohort are shown in Table 1. The average age of subjects was 51.5 (SD 17.7) years. The majority of subjects were from the white ethnic group (75.7%), and their average energy intakes were 1586.9 kcal/d (SD 465.6). The 24 h urine samples were collected from 98 males and 120 females, whereas plasma samples were from 89 males and 106 females. The plant-rich dietary scores, including DASH, MIND, O-MED, A-MED, and PDI (along with hPDI and uPDI), among the overall population are also described in Table 1. The score range was as following: 8–40 (DASH), 0–15 (MIND), 0–9 (O-MED and A-MED), and 18–90 (PDI, hDPI, and uPDI). The correlation between dietary scores is reported in Supplemental Fig. 1. Correlation coefficients ranged from − 0.60 to 0.85, among which the strongest correlation was observed between A-MED and O-MED (*r* = 0.85), whereas the weakest correlation was found between PDI and uPDI (*r* = 0.10).

ABP cohort & TwinsUK cohort: The overall characteristics of the participants in the ABP cohort and TwinsUK cohort are shown in Supplemental Table 1. The average age of subjects was 55.8 (SD 8.7) years in the ABP cohort and 62.0 (SD 9.9) in the TwinsUK cohort. Most of the subjects were from the white ethnic group (ABP: 79.5% and TwinsUK: 99.0%), and their average energy intakes were 1,681.3 kcal/d (SD 487.9) and 1,782.5 kcal/d (SD 554.3) in ABP and TwinsUK study, respectively. The 24 h urine samples in the ABP cohort were collected from 45 males and 43 females, and the spot urine samples (*n* = 198) in the TwinsUK cohort were all from females.

**Table 1:** Characteristics of the Study Population of the POLYNTAKE Cohort

Characteristics	Men	Women	Total
	Mean (SD)/ *n* (%)	Mean (SD)/ *n* (%)	Mean (SD)/ *n* (%)
Age (years) (mean, SD)	52.3 (16.9)	50.8 (18.3)	51.5 (17.7)
Ethnicity (n, %)			
White	72 (73.5)	93 (77.5)	165 (75.7)
Black	7 (7.1)	6 (5.0)	13 (6.0)
Asian	12 (12.2)	20 (16.7)	32 (14.7)
Mixed	7 (7.1)	1 (0.8)	8 (3.7)
Energy intake (kcal/d) (mean, SD)	1724.4 (486.0)	1474.5 (417.6)	1586.9 (465.6)
Sample (n, %)			
24 h urine	98 (45.0)	120 (55.0)	218
Plasma *	89 (45.6)	106 (54.4)	195
Plant-rich dietary scores (mean, SD)			
DASH	23.8 (5.4)	26.4 (4.3)	25.3 (5.0)
MIND	8.1 (1.5)	9.0 (1.6)	8.6 (1.6)
O-MED	4.2 (1.9)	4.5 (1.8)	4.4 (1.9)
A-MED	4.3 (2.0)	4.6 (2.1)	4.5 (2.1)
PDI	52.1 (5.9)	50.6 (6.2)	51.3 (6.1)
hPDI	51.8 (8.1)	55.6 (7.7)	53.9 (8.1)
uPDI	51.2 (7.0)	49.4 (6.9)	50.2 (7.0)

*: A total of 195 participants had plasma samples additionally collected. DASH, Dietary Approaches to Stop Hypertension; MIND, Mediterranean-DASH Intervention for Neurodegenerative Delay; O-MED, Original Mediterranean Score; A-MED, Amended Mediterranean Score; PDI, Plant-based Diet Index; hPDI, Healthy Plant-based Diet Index; uPDI, unhealthy Plant-based Diet Index

### Plant-rich dietary patterns and urinary metabolites

**Fig. 2:**
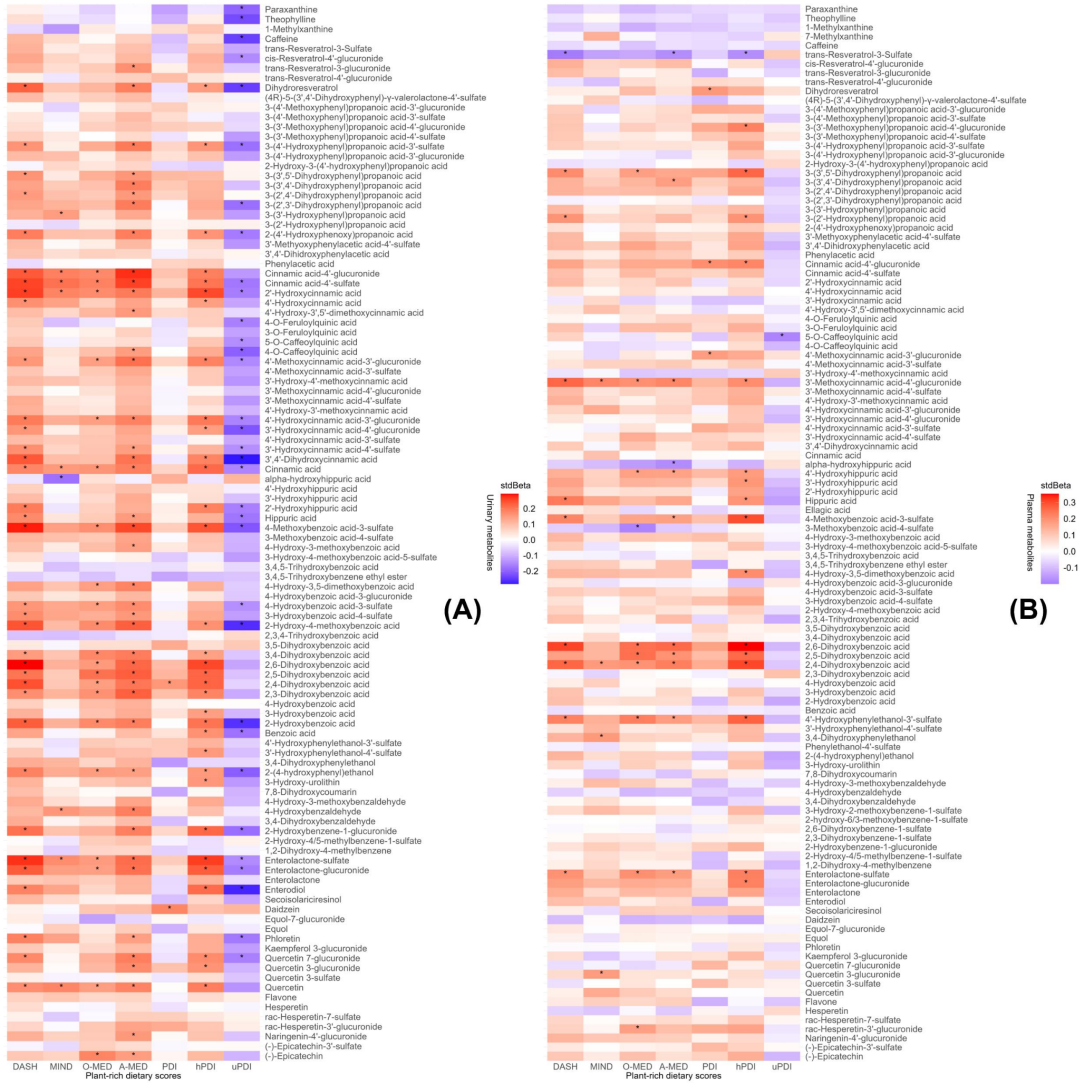
Association between (**A**) urinary and (**B**) plasma metabolites and plant-rich dietary scores. The heatmap was plotted according to the standardized regression coefficients (stdBeta). The colour scale indicates the effect (stdBeta) of each urinary or plasma metabolite on plant-rich dietary scores. Red and blue illustrate positive and negative effects, and colour intensity represents the degree of effect. The asterisks showed significance (*: FDR-adjusted *p* < 0.05). DASH, Dietary Approaches to Stop Hypertension; MIND, Mediterranean-DASH Intervention for Neurodegenerative Delay; O-MED, Original Mediterranean Score; A-MED, Amended Mediterranean Score; PDI, Plant-based Diet Index; hPDI, Healthy Plant-based Diet Index; uPDI, unhealthy Plant-based Diet Index. The associations were adjusted for energy intake

The association between 108 urinary metabolites and plant-rich dietary patterns is shown in Fig. 2(A). The significant standardized regression coefficients (and 95% CI) between urinary metabolite levels and all plant-rich dietary scores except uPDI were all positive, ranging from 0.14 (0.01, 0.27) for 3,4-dihydroxybenzoic acid (protocatechuic acid) and hPDI to 0.29 (0.16, 0.42) for 2,6-dihydroxybenzoic acid and DASH, except for the stdBeta for alpha-hydroxyhippuric acid and MIND, which is negative (-0.18 (-0.31, -0.05)). Negative associations were found between uPDI and 33 urinary metabolites from each class and most of them being phenolic acids (*n* = 21), including eleven cinnamic acids, five hydroxybenzoic acids, three phenylpropanoic acids, and two hippuric acids (all FDR-adjusted *p* < 0.05). Among the dietary scores that positively linked with metabolites, A-MED correlated with the highest number of metabolites (*n* = 42), followed by DASH, hPDI, and O-MED with 33, 33, and 22 metabolites associated, respectively, whereas MIND (*n* = 8) and PDI (*n* = 2) ranked lowest (all FDR-adjusted *p* < 0.05). Phenolic acids contributed the most to the positive associations with dietary scores, including 29 individual metabolites associated with A-MED, 26 with DASH, 22 with hPDI, 17 with O-MED, six with MIND, and one with PDI (all FDR-adjusted *p* < 0.05).

The association between 116 plasma metabolites and plant-rich dietary patterns is shown in Fig. 2(B). Among the scores that positively linked with plasma metabolites, hPDI was associated with the highest number of metabolites (*n* = 16), followed by DASH, A-MED, and O-MED with ten, nine, and nine metabolites associated, respectively. Similar to the urinary metabolites, in plasma, MIND and PDI also ranked lowest among all dietary scores, with four and three metabolites positively associated (all FDR-adjusted *p* < 0.05). In plasma, phenolic acids also contributed to the highest number of positive associations with the dietary scores, including 13 individual metabolites associated with hPDI, seven with DASH and A-MED, six with O-MED, and two with MIND and PDI (all FDR-adjusted *p* < 0.05). The significant positive stdBeta (and 95% CI) ranged from 0.17 (0.04, 0.30) for 4’-hydroxyhippuric acid and A-MED to 0.35 (0.22, 0.48) for 2,6-dihydroxybenzoic acid and hPDI. Negative associations were found between uPDI and 5-*O*-caffeoylquinic acid (chlorogenic acid), trans-resveratrol-3-sulfate and DASH, A-MED and hDPI, 3-methoxybenzoic acid-4-sulfate (vanillic acid-4-sulfate) and O-MED, alpha-hydroxyhippuric acid and A-MED with the stdBeta ranging from − 0.20 (trans-resveratrol-3-sulfate and DASH) to -0.17 (trans-resveratrol-3-sulfate and A-MED, FDR-adjusted *p* < 0.05). No significant associations were found between methylxanthine metabolites and dietary patterns (FDR-adjusted *p* > 0.05).

### Development and validation of metabolic signatures for plant-rich dietary patterns

**Fig. 3:**
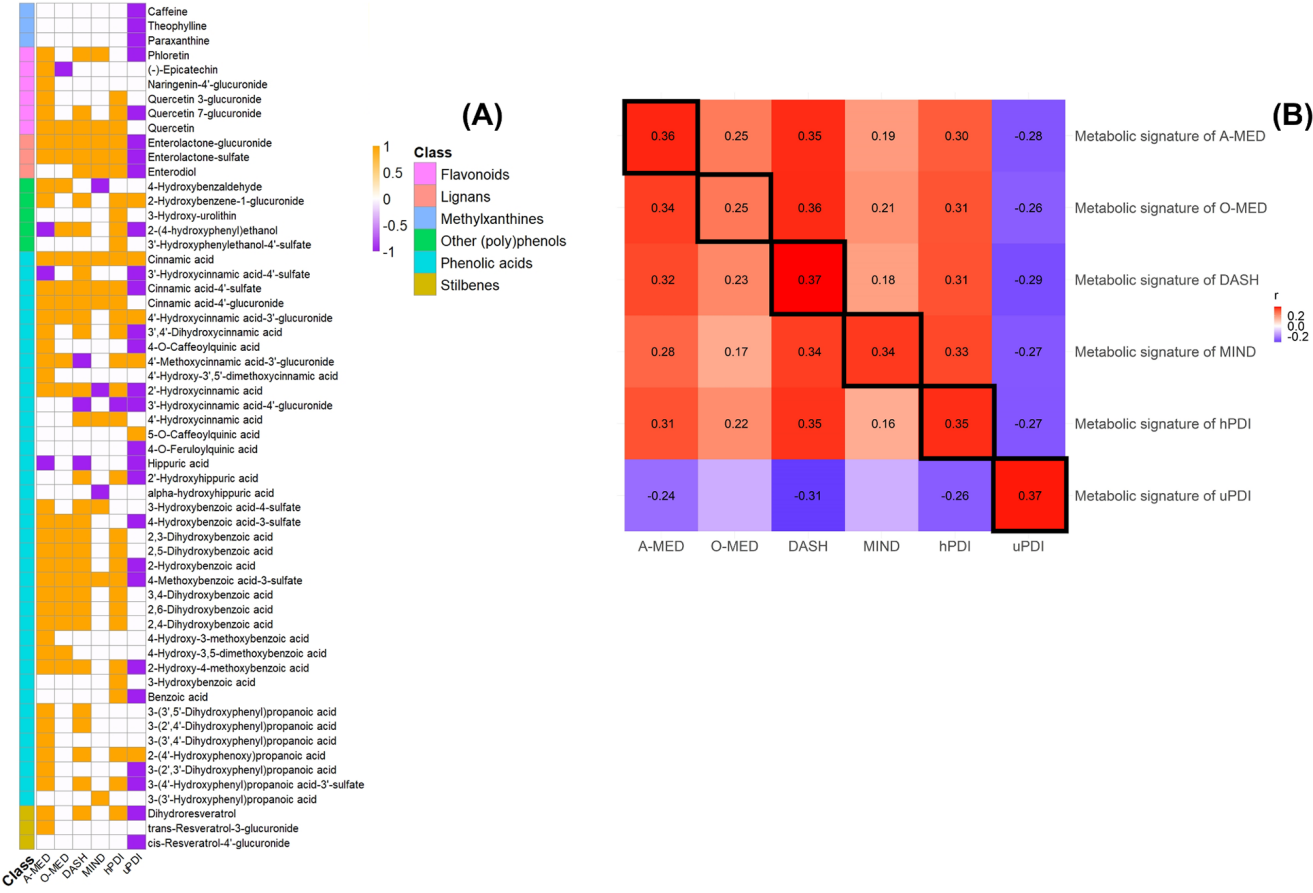
(**A**) Selected metabolites for each dietary pattern metabolic signature and (**B**) Correlation matrix between dietary patterns and metabolic signatures from the derivation set in the POLYNTAKE cohort with 24 h urine sample (*n* = 218). (**A**) The overlapping and distinct sets of the selected metabolites from each plant-rich dietary pattern. Yellow, purple, and white illustrated significant positive, negative, and non-significant associations in each dietary score. (**B**) The dietary scores were measured by FFQ. The metabolic signatures were derived based on the selected metabolites that were significantly associated with each plant-rich dietary score. The colour scale indicated the Spearman correlation coefficient between plant-rich dietary patterns and metabolic signatures. Red and blue illustrated positive and negative correlations and colour intensity represented the degree of the coefficient. The correlation with significance has listed the coefficient (FDR-adjusted, *p* < 0.05). DASH, Dietary Approaches to Stop Hypertension; MIND, Mediterranean-DASH Intervention for Neurodegenerative Delay; O-MED, Original Mediterranean Score; A-MED, Amended Mediterranean Score; hPDI, Healthy Plant-based Diet Index; uPDI, unhealthy Plant-based Diet Index

To establish a metabolic signature that captures multiple characteristics of a dietary pattern, the 24 h urine sample, which exhibited more significant metabolites with plant-rich dietary patterns than the plasma sample, was chosen. The detailed parameters of the selected metabolites for each plant-rich dietary score were listed in Supplemental Tables 1 to 6. No metabolic signature was summarized for PDI due to the limited significant associated metabolites (*n* = 2). The metabolic signatures for A-MED, O-MED, DASH, MIND, hPDI, and uPDI consisted of 42, 22, 35, 15, 33, and 33 predictive metabolites, respectively (Fig. 3(A), Supplemental Tables 1 to 6).

Figure 3(A) shows the overlapping and distinct sets of metabolites that were associated with plant-rich dietary patterns A-MED, O-MED, DASH, MIND, hPDI, and uPDI, and were made up predominantly of phenolic acids (*n* = 38), including 14 cinnamic acids, 14 hydroxybenzoic acids, 7 phenylacetic acids, and 3 hippuric acids. Besides phenolic acids, 3 methylxanthines, 6 flavonoids, 3 lignans, 3 stilbenes, 2 tyrosols, 1 benzaldehyde, hydroxycoumarin, and benzene diol and triol were also shown significant associations with the dietary scores. Six metabolites were included in all signatures, including 2 lignans (enterolactone-glucuronide, and enterolactone-sulfate), and 4 phenolic acids (cinnamic acid, cinnamic acid-4’-sulfate, 2’-hydroxycinnamic acid, and 4-methoxybenzoic acid-3-sulfate). Supplemental Fig. 2 listed the percentage of classes and subclasses of metabolites included on each metabolic signature. Hydroxybenzoic acids and cinnamic acids were the subclasses of phenolic acids which accounted for the dominant proportion in the composition of each signature (hydroxybenzoic acids, A-MED: 28.6%, O-MED: 45.5%, DASH: 28.6%, hPDI: 30.3%; cinnamic acids, DASH: 28.6%, MIND: 33.3%, uPDI: 33.3%).

The scatter plot in Supplemental Fig. 3 identified the significant positive relationship between each dietary score in quintiles and the corresponding metabolic signature (p for trend < 0.05). In Fig. 3(B), a Spearman correlation was implemented to assess the statistical correlation between each dietary pattern and the corresponding metabolic signature in the POLYNTAKE cohort using 24 h urine samples. The strongest correlation was found between uPDI, DASH, and their metabolic signatures (0.37 (0.25, 0.48)), followed by A-MED (0.36 (0.24, 0.47)), hPDI (0.35 (0.23, 0.46)), and MIND (0.34 (0.22, 0.45)). O-MED and its corresponding metabolic signature ranked lowest (0.25 (0.12, 0.37)) (all FDR-adjusted *p* < 0.05). For comparison, the highest coefficients of the association between each dietary score and individual urinary metabolites were 0.28 (0.15, 0.40), 0.21 (0.08, 0.33), 0.29 (0.16, 0.42), 0.21 (0.08, 0.34), 0.26 (0.14, 0.39) and − 0.28 (-0.40, -0.16) for A-MED (cinnamic acid-4’-sulfate), O-MED (2,5-dihydroxybenzoic acid), DASH (2,6-dihydroxybenzoic acid), MIND (cinnamic acid-4’-sulfate), hPDI (enterolactone-sulfate) and uPDI (caffeic acid), respectively. The correlations between metabolites and dietary patterns were stronger after assembling metabolites into a composite metabolic signature (Supplemental Table 8).

The validation cohort employed 3 types of samples across three cohorts, including the ABP cohort with 24 h urine samples, the POLYNTAKE cohort with plasma samples, and the TwinsUK cohort with spot urine samples in Supplemental Fig. 4. A significant positive Spearman correlation was found between each dietary pattern and their metabolic signatures across cohorts (all FDR-adjusted *p* < 0.05), except for the suggestive significance between uPDI and its signature in the POLYNTAKE cohort with plasma sample (0.13 (-0.01, 0.27), FDR-adjusted *p* = 0.05), and MIND with its signature in the TwinsUK cohort with spot urine (0.13 (-0.01, 0.26), FDR-adjusted *p* = 0.06).

The strongest correlation across the 3 validation cohorts was found between A-MED and its metabolic signature (ABP cohort: 0.40 (0.21, 0.56), FDR-adjusted *p* < 0.01), followed by the other 4 dietary scores with their metabolic signatures in the ABP cohort (O-MED, 0.36 (0.16, 0.53), hPDI, 0.35 (0.16, 0.53) and MIND, 0.35 (0.15, 0.52), DASH, 0.33 (0.13, 0.51), FDR-adjusted *p* < 0.05), whereas the lowest correlation was found in TwinsUK cohort with A-MED, DASH and O-MED lower than 0.20 (A-MED: 0.15 (0.02, 0.29), DASH: 0.16 (0.03, 0.30), O-MED: 0.16 (0.02, 0.29), FDR-adjusted *p* < 0.05). The correlation range of the ABP cohort ranked highest from 0.28 (0.07, 0.46) to 0.40 (0.21, 0.56), followed by POLYNTAKE cohort with plasma ranked middle from 0.21 (0.07, 0.34) to 0.30 (0.17, 0.43), and TwinsUK cohort lowest from 0.15 (0.01, 0.29) to 0.24 (0.10, 0.37) (all FDR-adjusted *p* < 0.05).

### Agreements between plant-rich dietary patterns and metabolic signatures

**Fig. 4:**
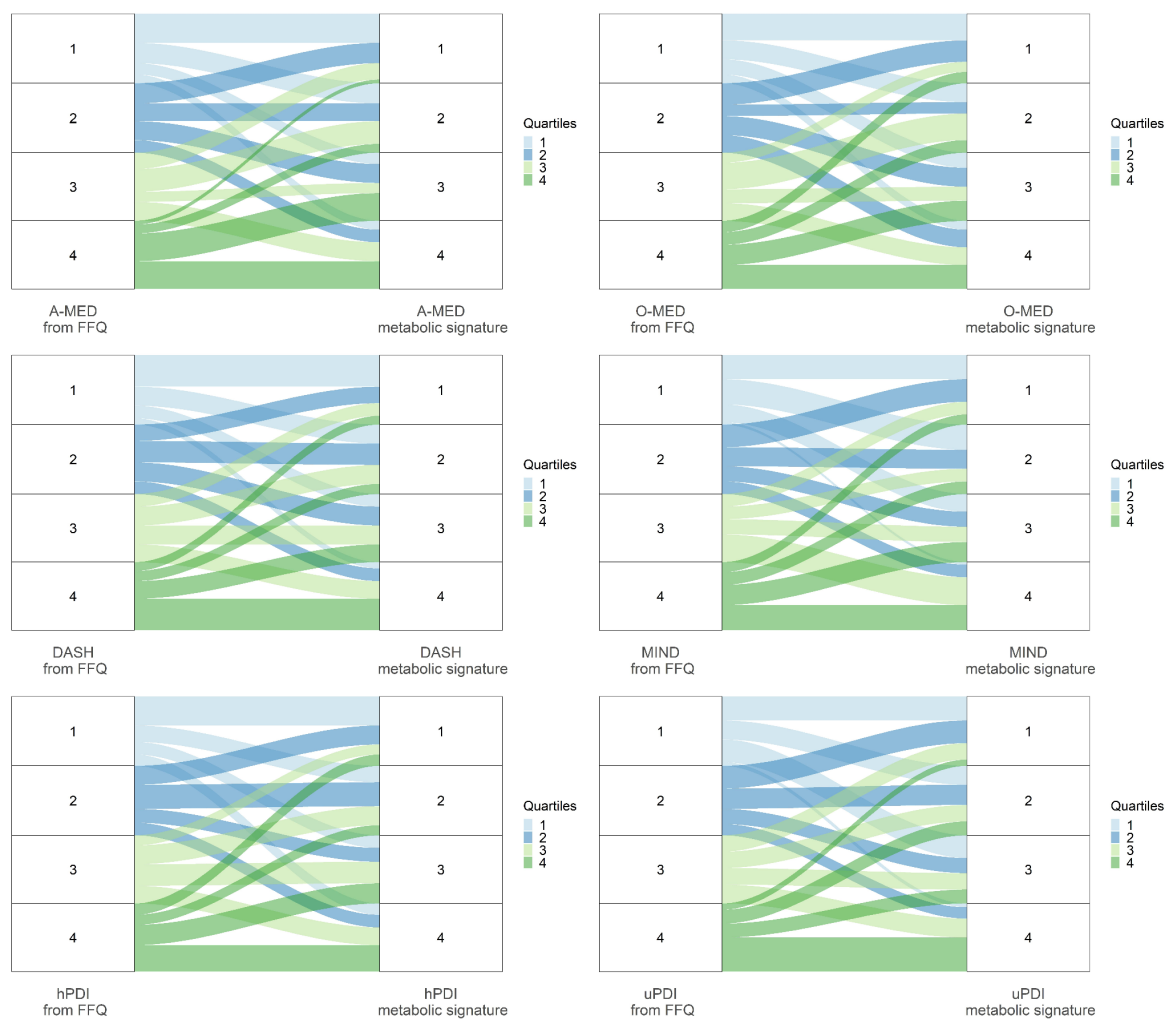
Agreements between plant-rich dietary patterns and their respective metabolic signatures in ranking participants into quartiles: The POLYNTAKE Study (24 h urine samples). DASH, Dietary Approaches to Stop Hypertension; MIND, Mediterranean-DASH Intervention for Neurodegenerative Delay; O-MED, Original Mediterranean Score; A-MED, Amended Mediterranean Score; hPDI, healthy Plant-based Diet Index; uPDI, unhealthy Plant-based Diet Index

The agreements between a *priori* plant-rich dietary patterns estimated from FFQs and metabolic signatures derived from 24 h urine samples in the POLYNTAKE cohort when ranking participants into quartiles are shown in Fig. 4. The two methods were comparable in differentiating participants into high and low adherence to the dietary patterns A-MED, O-MED, DASH, MIND, hPDI, and uPDI, with 77.5%, 72.5%, 77.1%, 75.2%, 75.2%, 72.9% of participants ranked into the same quartile or adjacent quartile, and only 4.6%, 7.3%, 5.5%, 4.6%, 8.3%, 3.7% ranked into the opposite quartile (the 1st and 4th quartile), respectively in Supplemental Table 9.

The agreements between plant-rich dietary patterns deriving from FFQs and metabolic signatures when ranking participants into quartiles from the TwinsUK cohort (spot urine), POLYNTAKE cohort (plasma), and ABP cohort (24 h urine) are shown in Supplemental Fig. 5 to 7. Among the various dietary patterns across different cohorts and samples, hPDI showed the best comparable performance between the dietary score from FFQs and the metabolic signature from the samples in differentiating participants in high and low adherence to the dietary pattern, with 75.9%, 70.3%, and 76.1% of participants ranked into the same quartile or adjacent quartile, respectively in Supplemental Table 9.

### Metabolic signatures and animal-based food items

**Fig. 5:**
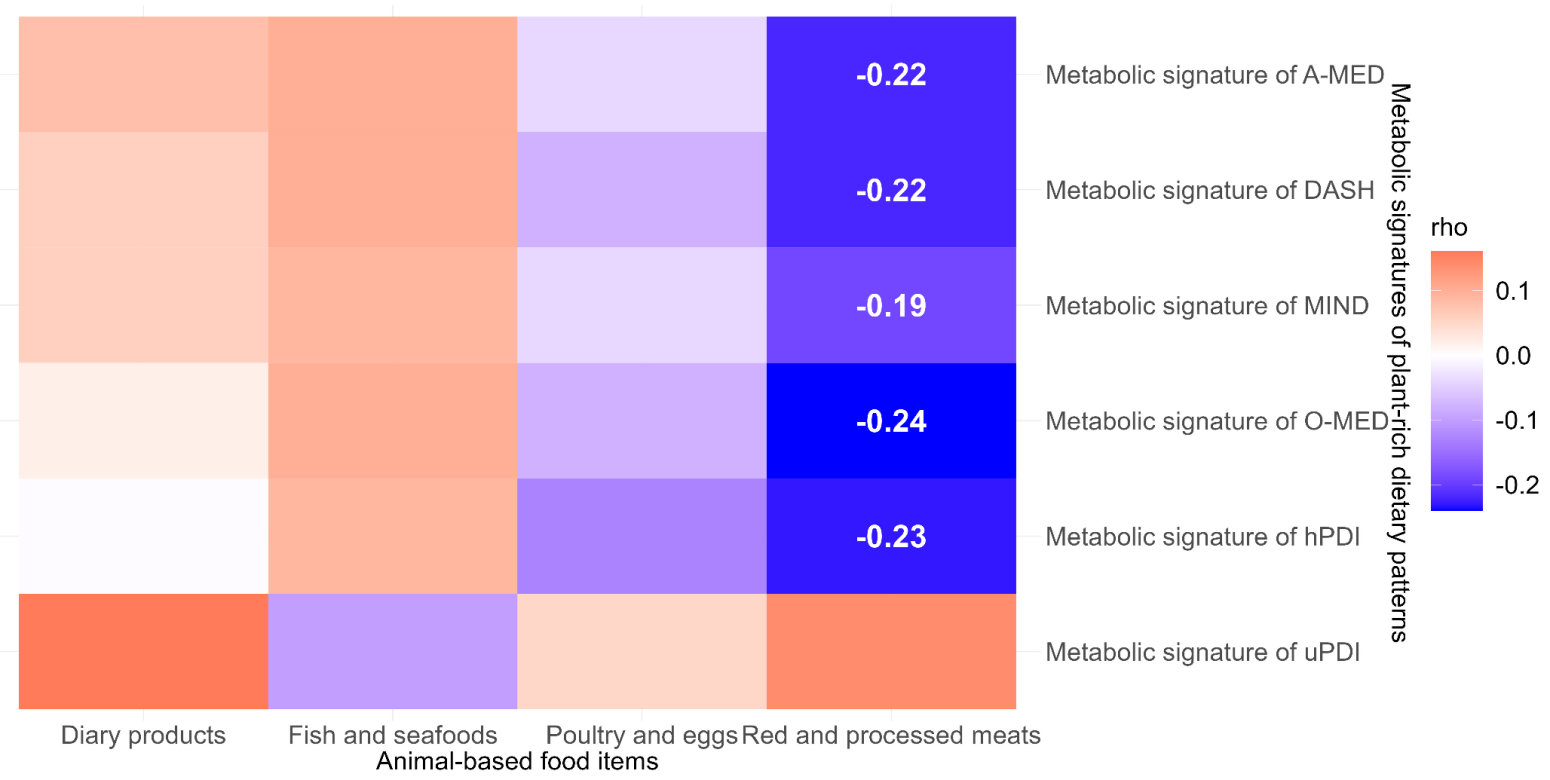
The Spearman correction between the metabolic signatures and animal-based food items. Red and blue illustrated respectively positive and negative correlations and colour intensity represented the degree of the coefficient. The correlation with significance were listed the coefficient (FDR-adjusted, *p* < 0.05), DASH, Dietary Approaches to Stop Hypertension; O-MED, Original Mediterranean Score; A-MED, Amended Mediterranean Score; MIND, Mediterranean-DASH Intervention for Neurodegenerative Delay; hPDI, healthy Plant-based Diet Index; uPDI, unhealthy Plant-based Diet Index

The relationships between the animal-based food items and the metabolic signatures from the derivation dataset are shown in Fig. 5. Red and processed meat negatively significantly correlated with the metabolic signatures of O-MED, A-MED, DASH, MIND, and hPDI, with rho ranging from − 0.19 (MIND, 95% CI: -0.31, -0.06) to -0.24 (O-MED, 95% CI: -0.36, -0.11), FDR-adjusted *p* < 0.05). No correlations were found for the other animal-based food items.

## Discussion

Here, we report the development and evaluation of metabolic signatures that measure adherence to a series of frequently applied plant-rich dietary patterns based on targeted metabolomics data of plant food metabolites in a population-based cohort of healthy adults. The metabolic signature combining a set of metabolites reflected a stronger correlation with the dietary patterns than a single biomarker and exhibited agreements in differentiating participants into high and low scores. Dietary patterns and metabolic signatures were also significantly correlated in the internal and external validation cohorts using different sample types. The result indicated the potential application of assembling a set of plant food metabolites as a composite marker of plant-rich dietary quality.

Enterolactone metabolites (enterolactone-glucuronide and enterolactone-sulfate) were significantly associated with all dietary patterns, and they are one of the most robust metabolites across all the plant-rich dietary patterns. It is the major microbial-derived metabolite of lignans, which are present in many plant foods, for instance, seeds, vegetables, fruits, legumes, wholegrains, extra virgin olive oil and non-alcoholic beverages such as tea and coffee [32], with the fibre-rich wholegrain outer layer being one of the main sources of dietary lignans [32, 33]. This wide distribution in plant-based products indicates its potential as a robust biomarker of plant-rich dietary patterns. The significant correlation of urinary enterolactone with plant-rich dietary scores has been reported in previous studies, including A-MED, DASH, and Alternative Healthy Eating Index (AHEI-2010) [34, 35] with a measurable concentration (0.006–27.6 µmol·L^− 1^) [35]. Therefore, enterolactone has potential as a reliable and robust biomarker for habitual plant-rich diet consumption. The metabolic signature of each dietary pattern had a stronger correlation than any single biomarker, including enterolactone, indicating that the combination of metabolites as a composite metabolic signature may be a better approach to capture the characteristics of a plant-rich diet.

Phenolic acids, as secondary plant metabolites and (poly)phenol gut microbial derivatives, are widely found in the plant kingdom, for instance, vegetable, fruits, especially berries, and beverages (coffee, tea, and wine) [36]. Coherently, there were 38 phenolic acid metabolites associated with various dietary patterns, in particular hydroxy derivates subclasses, including benzoic (*n* = 14), cinnamic (*n* = 14), phenylacetic (*n* = 7) and hippuric acids (*n* = 3) [36]. Hydroxybenzoic acids and hydroxycinnamic acids are the two main well-known subclasses of phenolic acids. Hydroxycinnamic acids are commonly found as simple esters, while hydroxybenzoic acids are mainly found in glycosylated form [36, 37]. Hydroxycinnamic acids include coumaric, ferulic, sinapic, caffeic and chlorogenic acids [37], and metabolites belonging to these subclasses were associated with the plant-rich dietary patterns, especially cinnamic acid, cinnamic acid-4’-sulfate, and 2’-hydroxycinnamic acid with a robust linkage across all diets. In the subclass of hydroxybenzoic acids, 4-methoxybenzoic acid-3-sulfate (isovanillic acid-3-sulfate), a compound which is a metabolite of many (poly)phenols including anthocyanins, for instance, present in berries [38], or plums [39], was also identified as a robust biomarker, indicating (poly)phenol-rich foods widely abundant in all plant-rich dietary patterns. Several studies have suggested that gut microbial-derived metabolites might be better biomarkers for assessing habitual diet than the parent species due to their restricted absorption and metabolization in the human body [35]. However, parent compounds are less variable and potentially more reliable compared with the gut microbial-derived metabolites. Since these coumaric acids and many other phenolic acids are also presented in food items, it is hard to distinguish whether they are microbial metabolites or parent compounds. Due to the high variability between individuals, the gut microbial-derived metabolites may reflect better human bioavailability and exposure than accurate habitual food intake.

Other classes of urinary metabolites were also found to be significantly associated to some of the plant-rich dietary patterns investigated here, however, none were robustly associated across all of them. Flavonoids represent a large class of (poly)phenol metabolites in the UK diet, including flavan-3-ols (epicatechin, mainly from tea), flavanones (naringenin-4’-glucuronide, mainly from citrus fruit), flavonols (quercetin, quercetin-3-glucuronide, and quercetin-7-glucuronide, mainly from tea, apples, and onions) [40], and dihydrochalcones (phloretin, mainly from apple) [41]. In the present study, A-MED was significantly associated with all these flavonoid metabolites, and the other patterns were associated with some of them. The stilbene resveratrol is commonly found in red grapes, peanuts, and red wine, although in very low concentrations [42]. In the present study, a positive association was found between the metabolites cis-resveratrol-4’-glucuronide, dihydroresveratrol, and trans-resveratrol-3-glucuronide with A-MED, DASH, and hPDI, reflecting the inclusion of alcohol (including red wine) and fruits (including grapes) in these dietary scores. Caffeine and its metabolites, paraxanthine and theophylline, are methylxanthines found in chocolate, cocoa, and beverages such as coffee and tea [43]. Our findings indicate negative associations between uPDI and these metabolites, consistent with the negative scoring of tea and coffee in the uPDI algorithm. Besides traditional sources like tea and coffee, energy drinks and dietary supplements have emerged as significant dietary sources of caffeine in recent years [44]. Currently, the lack of mandatory caffeine labelling, shifting classification of products from dietary supplements to beverages, and inconsistent definitions of energy products complicate the estimation of their dietary intake [44]. Caffeine intake derived from energy products and supplements is often excluded from dietary pattern calculations. While plant-derived supplements are less prevalent than plant-rich foods, future dietary research should integrate data from these sources to more accurately estimate caffeine intake. Metabolites related to the intake of olive oil (i.e., 3’-hydroxyphenylethanol-4’-sulfate (hydroxytyrosol-4’-sulfate) and 2-(4-hydroxyphenyl)ethanol (tyrosol)) [35] were positively associated with MED diet, DASH, and hPDI. Although olive oil was not included in the DASH scoring system, this indicates that olive oil-related metabolites might be reliable predictors of healthy habitual diets. Urolithins, as gut microbial metabolites of ellagitannins, were found to be associated with multiple ellagitannin-rich foods, such as pomegranates, berries, and walnuts [45]. Here, a positive association was only found between urolithin B and hPDI. However, it is worth mentioning that we did not measure the most abundant urolithin phase II metabolites, such as urolithin A glucuronide and sulfate, due to the difficulty in obtaining such standards commercially.

The metabolic signature of the Mediterranean diet has been proposed in several studies [13, 15]. For instance, a signature comprising 67 out of 302 metabolites was derived from the Spanish PREDIMED trial [13], while 66 out of 175 metabolites assembling a signature of MDS was identified in the Fenland Study in the UK [15]. In the present study, 42 and 22 candidate metabolites out of 108 metabolites were selected to establish the signatures of A-MED and O-MED. Compared with O-MED, the signature of A-MED performed better in differentiating participants into high and low adherence to the diet and showed a stronger correlation with the dietary pattern, especially the moderate correlation found in the ABP validation cohort (*r* = 0.40). This difference may be due to the different food groups included in the scoring system. A-MED does not include dairy, starchy vegetables, and refined grains, while it includes wholegrains and pure fruit juice in comparison with the original MED score [46]. The plant food metabolites showed a stronger correlation with plant-based food items, such as fruit juice than animal-derived food, such as dairy. This is also reflected in the metabolic signatures, with naringenin-4’-glucuronide (mainly coming from citrus fruit and juice) and phloretin (mainly coming from apple and juice) being associated with A-MED but not O-MED. The correlation coefficient of the A-MED signature (*r* = 0.16–0.40) was similar to past research (*r* = 0.28–0.37 [13], *r* = 0.43 [15]), despite the lower metabolite number in our study. The higher number of metabolites in other signatures may be attributed to animal-sourced food items, for instance, acylcarnitines (meat) and phospholipids (fish) [31].

The signature of the DASH diet exhibited a similarly good performance as A-MED, with 35 significantly associated metabolites, and the highest correlation with the dietary pattern in the derivation cohort. This result may be attributed to the wide range of its predefined food profile, which included vegetables, fruits, and wholegrains, and limited the intake of processed meat, fat, refined grains, and alcohol [5]. However, the MIND signature did not achieve a satisfying result in ranking participants into quartiles, and showed a weaker correlation with the MIND dietary pattern. The MIND score was developed based on two randomized trials investigating the effect of MED and DASH diets on dementia [8]. Compared with other plant-rich patterns, the MIND diet narrowed the range of the predefined food profile by including specific food items, for instance, berries (instead of all types of fruits), beans (instead of legumes), cheese (instead of dairy products), and specific cooked-food, such as fast fried food. This restricted range may explain why it was hard to define an appropriate metabolic signature for the MIND diet (with only 15 predictive biomarkers) due to being too specific.

PDI was established to investigate associations between health outcomes and the gradual reduction in animal food intake and the increase in consumption of healthy plant-foods [6]. Three versions of this diet index were defined with different emphasis, including all plant food with a reduction in animal food (overall PDI), healthy plant food linked with beneficial health outcomes (hPDI), and unhealthy plant food linked with detrimental health outcomes (uPDI) [6]. Refined grains, potatoes, and sugar-sweetened beverages are positively linked to uPDI, whereas high-quality plant products, for instance, wholegrains, fruits, and vegetables, are positively linked to hPDI [24]. These dietary algorithms were substantiated by the large number of biomarkers that significantly correlated with hPDI (*n* = 33), inversely with uPDI (*n* = 33) and limited with PDI (*n* = 2). The signature of uPDI exhibited the strongest inverse correlation with dietary patterns in the derivation cohort, and hPDI showed the best agreement in ranking participants in comparison with other patterns in all validation cohorts. This result indicated that the metabolic signature of hPDI might be a promising predictive tool to identify the adherence of participants to a habitual plant-rich diet in large nutritional epidemiological studies. Compared with hPDI and uPDI, PDI was less specific and correlated with limited metabolites. Thus, no signature of PDI was established. Moreover, the varied performance of the three versions of the diet index [6] highlights the grading methodology as a reliable and logical approach for characterizing habitual plant-rich diets.

Metabolomics profiling represents a systematic analytical methodology for low-molecular-weight compounds in multiple biofluids, among which spot or 24 h urine, serum, and plasma samples are the most widely used sample types for biomarker analysis [14]. Compared with plasma, urine samples contain more compounds deriving from food phytochemicals, and secondary metabolites produced from plants or fungi that are valuable biomarkers for assessing plant-rich dietary habits [47]. Most metabolites are excreted fast in urine and can be used as acute biomarkers of food intake [14]. Plasma contains more metabolically active compounds than urine, and lipid-soluble compounds are only found in plasma. In this work, the urine samples reflected better the adherence to plant-rich diets than fasting plasma samples in the POLYNTAKE cohort. Urine has become a popular choice in nutritional epidemiology studies [48], and 24 h urine is optimal as it is quantitative and can quantify a larger number of metabolites with different half-lives than spot urine. However it has the inconvenience of burdersome collection, which is not always possible in large epidemiological studies [48]. For instance, the excretion of proline betaine peaks a few hours after consumption, and its concentration declines to almost baseline after 24 h [49]. We found that the correlation coefficient between dietary patterns and metabolic signatures decreased from the internal cohort ABP with 24 h urine, to the POLYNTAKE with plasma, to the external TwinsUK cohort with spot urine. This result may be due to the different nature of the sample types discussed above and partially to the study population of the TwinsUK cohort, which consisted of older female participants [19], compared with the derivation cohort.

To note, the alluvial plots effectively differentiate between high and low scores but perform less well in depicting a gradient. This may be attributed to the relatively homogenous dietary habits of the study population, leaning towards the healthy end of the spectrum, and the inherently relative nature of these diet scores. Cross-classification analysis is commonly employed to validate agreements between two methods in energy and nutrient intake research [50]. The gross misclassification of more than 10% of subjects into the opposite quartiles illustrates a poor outcome, while more than 70% of subjects correctly classified into the same or adjacent quartiles indicate a satisfactory agreement [50]. Here, all agreements in the derivation and ABP validation datasets met these satisfactory criteria. However, some dietary patterns in the other two validation datasets slightly missed the standard, such as A-MED and MIND in the two datasets, DASH in the TwinsUK dataset, and uPDI in the POLYNTAKE cohort with plasma sample for the correctly classified criteria, and O-MED in the two datasets for opposite quartiles. The ABP dataset, a subcohort of the POLYNTAKE cohort, shares the same participants’ characteristics and biospecimens as the derivation dataset, serving as an internal validation dataset. Thus, the metabolic signature derived from the overall derivation dataset is highly applicable to the ABP cohort, yielding similar validation results in terms of correctly classified and opposite quartile percentages. Additionally, certain dietary patterns, such as A-MED, O-MED, hPDI, and uPDI, exhibited superior correctly classified percentages compared to the derivation dataset. In contrast, the TwinsUK cohort, an external dataset, differs in both participants characteristics and biospecimens. These variations in biofluids and participant characteristics in the external validation datasets likely contribute to the slight deviations from the criteria observed in dietary patterns other than hPDI. The hPDI algorithm prioritizes plant-based food components and aligns with our plant-derived metabolites, ensuring satisfactory agreement across all validation datasets despite variations in participant characteristics or biospecimens.

The plant-rich dietary patterns investigated in this study also included animal-derived food items, for instance, fish, poultry, and meat products, which are high in choline and carnitine. Previous studies indicated that the metabolic signatures of meat intake included multiple metabolites such as lipids, amino acids, and xenobiotics [31]. Our targeted metabolomic profiling assay did not include any biomarkers of meat or fish consumption, for instance, trimethylamine-N-oxide (TMAO) [14]. The disagreement in classification between dietary patterns and their respective metabolic signature when ranking participants into quartiles in the alluvial plots may be attributed to the lack of animal-derived food metabolites. In addition, negative correlations were found between red and processed meat and dietary patterns, except for uPDI. Although it is hard to untangle the origin of each of the metabolites, some metabolites could come from non-plant sources. These results strengthened the validity of the metabolic signatures, assuming that higher consumption of plant foods may indicate a lower consumption of red and processed meat. Further research is required to investigate whether adding these animal-derived biomarkers would increase the correlation between dietary patterns and signatures.

As strengths, though studies have identified several dietary pattern-associated metabolic signatures, this study is the first to develop metabolic signatures for evaluating adherence to a series of common plant-rich diet patterns. The measurement of a wide range of metabolites from different classes in plasma, spot, and 24-hour urine with authentic standards enabled us to capture an accurate, quantitative, and comprehensive profiling of the plant food-related metabolome [26]. The metabolite-based, machine-learning-derived signatures were reproducible among free-living healthy adults across various sample types. The reliability and robustness of the methodology allows potential applications in future research to measure adherence to plant-rich dietary patterns/habits in prospective cohorts and interventional studies.

Limitations of this work include (i) the observational nature of this study limited the exploration of causality; (ii) the use of self-reported methods and a limited set of predefined food groups for each dietary pattern coming from FFQs may introduce measurement bias; (iii) the targeted metabolomics assay did not emphasize non-plant-related molecules relevant to meat-related food items, for instance, acylcarnitines, phospholipids, amino acids and amines [15]. The assay platform will expand in the future to gain more insights into meat-related metabolites, and the signatures may benefit from additional meat-related metabolites. Moreover, the generalization of the findings is limited to healthy adults living in the UK, so future replication in both the UK and other countries with a larger population is needed, including further examination of associations with health outcomes.

## Conclusion

In conclusion, a set of 6 metabolic signatures was developed and evaluated based on three UK-based cohorts to measure adherence to plant-rich dietary patterns. Phenolic acids and enterolactone metabolites, as gut microbial-derived compounds, played an important role in the establishment of the signatures. Agreements were found between dietary patterns and their corresponding signatures when ranking participants into quartiles, highlighting the value of the metabolic signature as a reliable marker of plant-rich dietary intake. Our findings highlight the potential of metabolomics profiling as a tool for objectively evaluating habitual dietary intake.

## Electronic supplementary material

Below is the link to the electronic supplementary material.

Supplementary Material 1

## Data Availability

The TwinsUK cohort data used in this study are held by the Department of Twin Research at King’s College London. The data can be released to bona fide researchers using our normal procedures overseen by the Wellcome Trust and its guidelines as part of our core funding (https://twinsuk.ac.uk/resources-for-researchers/access-our-data/).
