# Correction: Development of metabolic signatures of plant-rich dietary patterns using plant-derived metabolites

**DOI:** 10.1007/s00394-025-03684-z

**Published:** 2025-07-15

**Authors:** Yong Li, Yifan Xu, Melanie Le Sayec, Tim D. Spector, Claire J. Steves, Cristina Menni, Rachel Gibson, Ana Rodriguez-Mateos

**Affiliations:** 1 Department of Nutritional Sciences, School of Life Course and Population Sciences, Faculty of Life Sciences and Medicine, King’s College London, London, WC2R 2LS UK; 2 Department of Twin Research and Genetic Epidemiology, School of Life Course and Population Sciences, Faculty of Life Sciences and Medicine, King’s College London, London, WC2R 2LS UK; 3 Department of Pathophysiology and Transplantation, Università Degli Studi di Milano, Milan, 20122 Italy

**European Journal of Nutrition (2025) 64:29**

10.1007/s00394-024-03511-x

In the original version of this article, in Fig. 3, x axis was missing together with a couple of rows of the heatmap on the left and the label B was missing.

Figure 3, which previously appeared as

**Fig. 3:**
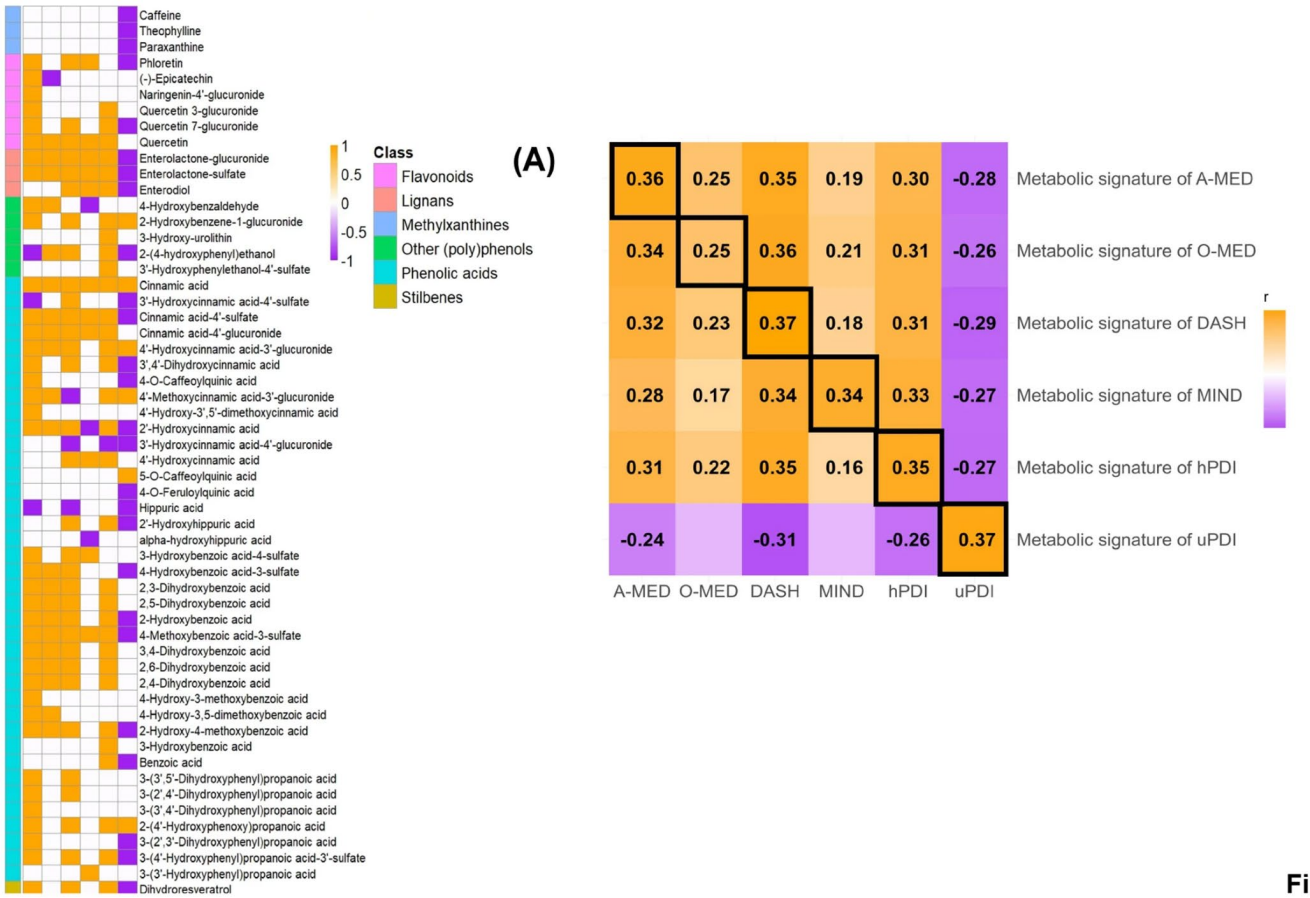
(A) Selected metabolites for each dietary pattern metabolic signature and (B) Correlation matrix between dietary patterns and metabolic signatures from the derivation set in the POLYNTAKE cohort with 24 h urine sample (*n* = 218). (A) The overlapping and distinct sets of the selected metabolites from each plant-rich dietary pattern. Yellow, purple, and white illustrated significant positive, negative, and nonsignificant associations in each dietary score. (B) The dietary scores were measured by FFQ. The metabolic signatures were derived based on the selected metabolites that were significantly associated with each plant-rich dietary score. The colour scale indicated the Spearman correlation coefficient between plant-rich dietary patterns and metabolic signatures. Red and blue illustrated positive and negative correlations and colour intensity represented the degree of the coefficient. The correlation with significance has listed the coefficient (FDR-adjusted, *p* ≤ 0.05). DASH, Dietary Approaches to Stop Hypertension; MIND, Mediterranean-DASH Intervention for Neurodegenerative Delay; O-MED, Original Mediterranean Score; A-MED, Amended Mediterranean Score; hPDI, Healthy Plant-based Diet Index; uPDI, unhealthy Plant-based Diet Index

but should have appeared as

**Fig. 3:**
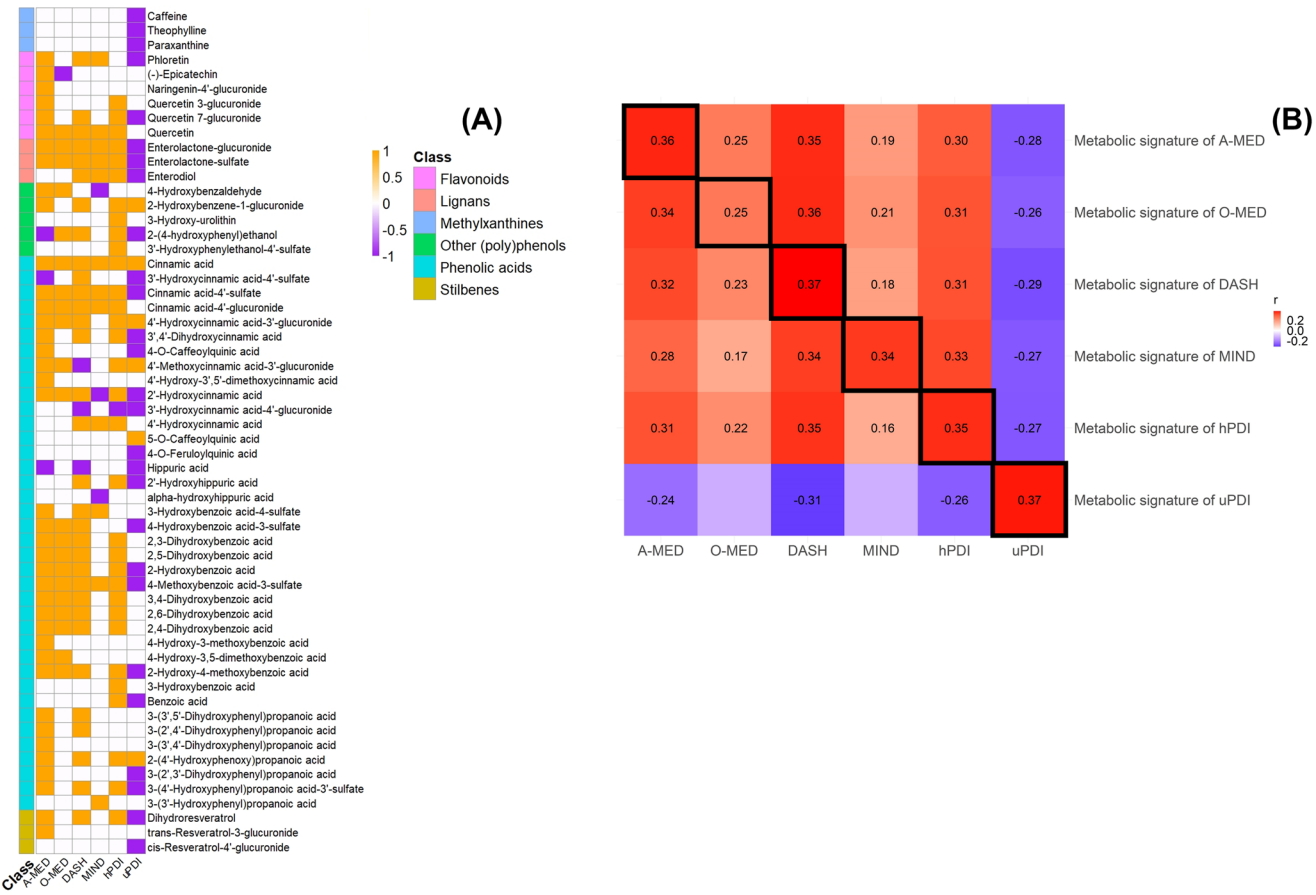
(A) Selected metabolites for each dietary pattern metabolic signature and (B) Correlation matrix between dietary patterns and metabolic signatures from the derivation set in the POLYNTAKE cohort with 24 h urine sample (*n* = 218). (A) The overlapping and distinct sets of the selected metabolites from each plant-rich dietary pattern. Yellow, purple, and white illustrated significant positive, negative, and nonsignificant associations in each dietary score. (B) The dietary scores were measured by FFQ. The metabolic signatures were derived based on the selected metabolites that were significantly associated with each plant-rich dietary score. The colour scale indicated the Spearman correlation coefficient between plant-rich dietary patterns and metabolic signatures. Red and blue illustrated positive and negative correlations and colour intensity represented the degree of the coefficient. The correlation with significance has listed the coefficient (FDR-adjusted, *p* ≤ 0.05). DASH, Dietary Approaches to Stop Hypertension; MIND, Mediterranean-DASH Intervention for Neurodegenerative Delay; O-MED, Original Mediterranean Score; A-MED, Amended Mediterranean Score; hPDI, Healthy Plant-based Diet Index; uPDI, unhealthy Plant-based Diet Index

The original article has been corrected.

