# Supplementary material for: Development of metabolic signatures of plant-rich dietary patterns using plant-derived metabolites
Source: Eur J Nutr. 2024 Nov 28;64(1):29. doi: 10.1007/s00394-024-03511-x (PMC11602792; doi:10.1007/s00394-024-03511-x)
Supplement: Supplementary file 1 — Supplementary Material 1 [file 394_2024_3511_MOESM1_ESM.docx]

**Development of metabolic signatures of plant-rich dietary patterns using plant-derived metabolites**

Yong Li ^1^, Yifan Xu ^1^, Melanie Le Sayec ^1^, Tim D Spector ^2^, Claire J Steves ^2^, Cristina Menni ^2,3^, Rachel Gibson ^1^, Ana Rodriguez-Mateos ^1, *^

^1^ Department of Nutritional Sciences, School of Life Course and Population Sciences, Faculty of Life Sciences and Medicine, King's College London, London WC2R 2LS, UK

yong.3.li@kcl.ac.uk (Y.L.); yifan.xu@kcl.ac.uk (Y.X.);

melanie.le_sayec@kcl.ac.uk (M.L.S); rachel.gibson@kcl.ac.uk (R.G.)

^2^ Department of Twin Research and Genetic Epidemiology, School of Life Course and Population Sciences, Faculty of Life Sciences and Medicine, King’s College London, London WC2R 2LS, UK

tim.spector@kcl.ac.uk (T.D.S.); claire.j.steves@kcl.ac.uk (C.J.S.); cristina.menni@kcl.ac.uk (C.M.)

^3^ Department of Pathophysiology and Transplantation, Università Degli Studi di Milano, 20122, Milan, Italy

cristina.menni@kcl.ac.uk (C.M.)

^*^ Author to whom correspondence should be addressed: ana.rodriguez-mateos@kcl.ac.uk

**Table of Contents**

Table S1. Characteristics of the ABP and TwinsUK cohort population.

Table S2. Overview of metabolites included for the dietary score A-MED.

Table S3. Overview of metabolites included for the dietary score O-MED.

Table S4. Overview of metabolites included for the dietary score DASH.

Table S5. Overview of metabolites included for the dietary score MIND.

Table S6. Overview of metabolites included for the dietary score hPDI.

Table S7. Overview of metabolites included for the dietary score uPDI.

Table S8. Comparison of correlation between metabolic signature and significant individual metabolites.

Table S9. Cross-validation between dietary pattern and corresponding metabolic signature in each dataset.

Figure S1. Correlation among plant-rich dietary scores.

Figure S2. The percentage of the metabolites in each metabolic signature from derivation set in the POLYNTAKE cohort with 24h urine sample (n = 218).

Figure S3. The correlations between metabolic signatures and plant-rich dietary patterns from derivation set in the POLYNTAKE cohort with 24h urine sample (n = 218).

Figure S4. The correlations matrix for plant-rich dietary patterns and metabolic signatures from validation dataset in the ABP study with 24h urine sample (n = 88), POLYNTAKE cohort with plasma sample (n = 195), and TwinsUK cohort with spot urine sample (n = 198).

Figure S5. Agreements between plant-rich dietary patterns and the metabolic signature in ranking participants into quartiles: the TwinsUK Study (spot urine, n = 198).

Figure S6. Agreements between plant-rich dietary patterns and the metabolic signature in ranking participants into quartiles: the POLYNTAKE Study (plasma, n = 195).

Figure S7. Agreements between plant-rich dietary patterns and the metabolic signature in ranking participants into quartiles: the ABP Study (24h urine, n = 88).

**Table S1. Characteristics of the ABP and TwinsUK cohort population**

| **Characteristics** | **ABP** | **TwinsUK** |
| --- | --- | --- |
| n | 88 | 200 |
| Age (years) (mean, SD) | 55.8 (8.7) | 62.0 (9.9) |
| Gender (n, %) |  |  |
| Male | 45 (51.1) | -- |
| Female | 43 (48.9) | 200 |
| Ethnicity (n, %) |  |  |
| White | 70 (79.5) | 198 (99.0) |
| Black | 8 (9.1) | 0 |
| Asian | 7 (8.0) | 0 |
| Mixed | 3 (3.4) | 2 (1.0) |
| Energy intake (kcal/d) (mean, SD) | 1681.3 (487.9) | 1782.5 (544.3) |
| Biosample (n, %) |  |  |
| 24h urine | 88 | -- |
| Spot urine | -- | 200 |
| Plant-rich dietary scores (mean, SD) |  |  |
| DASH | 24.7 (5.3) | 25.1 (4.3) |
| MIND | 8.4 (1.6) | 7.8 (1.5) |
| O-MED | 4.4 (1.9) | 4.5 (1.3) |
| A-MED | 4.5 (2.0) | 4.6 (1.9) |
| PDI | 52.7 (6.0) | 51.9 (7.0) |
| hPDI | 53.8 (9.3) | 53.3 (7.6) |
| uPDI | 54.9 (1.1) | 54.8 (7.4) |

DASH, Dietary Approaches to Stop Hypertension; MIND, Mediterranean-DASH Intervention for Neurodegenerative Delay; O-MED, Original Mediterranean Score; A-MED, Amended Mediterranean Score; PDI, Plant-based Diet Index; hPDI, Healthy Plant-based Diet Index; uPDI, unhealthy Plant-based Diet Index.

**Table S2. Overview of metabolites included for the dietary score A-MED**

| **Class** | **Subclass** | **Individual metabolite**  **(Common name)** | **Individual metabolite**  **(Recommended name)** | **Coefficient** |
| --- | --- | --- | --- | --- |
| Flavonoids | Dihydrochalcones | Phloretin | Phloretin | 0.017 |
| Flavonoids | Flavanols | (-)-Epicatechin | (-)-Epicatechin | 0.026 |
| Flavonoids | Flavanones | Naringenin-4'-glucuronide | Naringenin-4'-glucuronide | 0.002 |
| Flavonoids | Flavonols | Quercetin-3-glucuronide | Quercetin 3-glucuronide | 0.015 |
| Flavonoids | Flavonols | Quercetin-7-glucuronide | Quercetin 7-glucuronide | 0.040 |
| Flavonoids | Flavonols | Quercetin | Quercetin | 0.028 |
| Lignans | Lignans | Enterolactone-glucuronide | Enterolactone-glucuronide | 0.023 |
| Lignans | Lignans | Enterolactone-sulfate | Enterolactone-sulfate | 0.024 |
| Other (poly)phenols | Benzaldehydes | 4-Hydroxybenzaldehyde | 4-Hydroxybenzaldehyde | 0.038 |
| Other (poly)phenols | Benzene diols and triols | Catechol-1-glucuronide | 2-Hydroxybenzene-1-glucuronide | 0.000 |
| Other (poly)phenols | Tyrosols | Tyrosol | 2-(4-Hydroxyphenyl)ethanol | -0.004 |
| Phenolic acids | Cinnamic acids | Cinnamic acid | Cinnamic acid | 0.041 |
| Phenolic acids | Cinnamic acids | Caffeic acid-4'-sulfate | 3'-Hydroxycinnamic acid-4'-sulfate | -0.004 |
| Phenolic acids | Cinnamic acids | p-coumaric acid-4'-sulfate | Cinnamic acid-4'-sulfate | 0.041 |
| Phenolic acids | Cinnamic acids | p-coumaric acid-4'-glucuronide | Cinnamic acid-4'-glucuronide | 0.024 |
| Phenolic acids | Cinnamic acids | Caffeic acid-3'-glucuronide | 4'-Hydroxycinnamic acid-3'-glucuronide | 0.004 |
| Phenolic acids | Cinnamic acids | Caffeic acid | 3',4'-Dihydroxycinnamic acid | 0.014 |
| Phenolic acids | Cinnamic acids | Cryptochlorogenic acid | 4-O-Caffeoylquinic acid | 0.004 |
| Phenolic acids | Cinnamic acids | Isoferulic acid-3'-glucuronide | 4'-Methoxycinnamic acid-3'-glucuronide | 0.011 |
| Phenolic acids | Cinnamic acids | Sinapic acid | 4'-Hydroxy-3',5'-dimethoxycinnamic acid | 0.009 |
| Phenolic acids | Cinnamic acids | o-coumaric acid | 2'-Hydroxycinnamic acid | 0.030 |
| Phenolic acids | Hippuric acids | Hippuric acid | Hippuric acid | -0.001 |
| Phenolic acids | Hydroxybenzoic acids | Protocatechuic acid-4-sulfate | 3-Hydroxybenzoic acid-4-sulfate | 0.003 |
| Phenolic acids | Hydroxybenzoic acids | Protocatechuic acid-3-sulfate | 4-Hydroxybenzoic acid-3-sulfate | 0.004 |
| Phenolic acids | Hydroxybenzoic acids | 2,3-Dihydroxybenzoic acid | 2,3-Dihydroxybenzoic acid | 0.015 |
| Phenolic acids | Hydroxybenzoic acids | 2,5-Dihydroxybenzoic acid | 2,5-Dihydroxybenzoic acid | 0.029 |
| Phenolic acids | Hydroxybenzoic acids | 2-Hydroxybenzoic acid | 2-Hydroxybenzoic acid | 0.021 |
| Phenolic acids | Hydroxybenzoic acids | Isovanillic acid-3-sulfate | 4-Methoxybenzoic acid-3-sulfate | 0.045 |
| Phenolic acids | Hydroxybenzoic acids | Protocatechuic acid | 3,4-Dihydroxybenzoic acid | 0.010 |
| Phenolic acids | Hydroxybenzoic acids | 2,6-Dihydroxybenzoic acid | 2,6-Dihydroxybenzoic acid | 0.037 |
| Phenolic acids | Hydroxybenzoic acids | 2,4-Dihydroxybenzoic acid | 2,4-Dihydroxybenzoic acid | 0.030 |
| Phenolic acids | Hydroxybenzoic acids | Vanillic acid | 4-Hydroxy-3-methoxybenzoic acid | 0.009 |
| Phenolic acids | Hydroxybenzoic acids | Syringic acid | 4-Hydroxy-3,5-dimethoxybenzoic acid | 0.027 |
| Phenolic acids | Hydroxybenzoic acids | 2-Hydroxy-4-methoxybenzoic acid | 2-Hydroxy-4-methoxybenzoic acid | 0.090 |
| Phenolic acids | Phenylacetic acids | 3-(3',5'-Dihydroxyphenyl)propanoic acid | 3-(3',5'-Dihydroxyphenyl)propanoic acid | 0.021 |
| Phenolic acids | Phenylacetic acids | 3-(2',4'-Dihydroxyphenyl)propanoic acid | 3-(2',4'-Dihydroxyphenyl)propanoic acid | 0.005 |
| Phenolic acids | Phenylacetic acids | Dihydrocaffeic acid | 3-(3',4'-Dihydroxyphenyl)propanoic acid | 0.011 |
| Phenolic acids | Phenylacetic acids | 2-(4'-Hydroxyphenoxy)propanoic acid | 2-(4'-Hydroxyphenoxy)propanoic acid | 0.010 |
| Phenolic acids | Phenylacetic acids | 3-(2',3'-Dihydroxyphenyl)propanoic acid | 3-(2',3'-Dihydroxyphenyl)propanoic acid | 0.016 |
| Phenolic acids | Phenylacetic acids | Dihydrocaffeic acid-3'-sulfate | 3-(4'-Hydroxyphenyl)propanoic acid-3'-sulfate | 0.020 |
| Stilbenes | Stilbenes | Dihydroresveratrol | Dihydroresveratrol | 0.056 |
| Stilbenes | Stilbenes | trans-Resveratrol-3-glucuronide | trans-Resveratrol-3-glucuronide | 0.028 |

**Table S3. Overview of metabolites included for the dietary score O-MED**

| **Class** | **Subclass** | **Individual metabolite**  **(Common name)** | **Individual metabolite**  **(Recommended name)** | **Coefficient** |
| --- | --- | --- | --- | --- |
| Flavonoids | Flavanols | (-)-Epicatechin | (-)-Epicatechin | -0.006 |
| Flavonoids | Flavonols | Quercetin | Quercetin | 0.025 |
| Lignans | Lignans | Enterolactone-glucuronide | Enterolactone-glucuronide | 0.017 |
| Lignans | Lignans | Enterolactone-sulfate | Enterolactone-sulfate | 0.015 |
| Other (poly)phenols | Benzaldehydes | 4-Hydroxybenzaldehyde | 4-Hydroxybenzaldehyde | 0.012 |
| Other (poly)phenols | Tyrosols | Tyrosol | 2-(4-hydroxyphenyl)ethanol | 0.005 |
| Phenolic acids | Cinnamic acids | Cinnamic acid | Cinnamic acid | 0.021 |
| Phenolic acids | Cinnamic acids | p-coumaric acid-4'-sulfate | Cinnamic acid-4'-sulfate | 0.019 |
| Phenolic acids | Cinnamic acids | p-coumaric acid-4'-glucuronide | Cinnamic acid-4'-glucuronide | 0.002 |
| Phenolic acids | Cinnamic acids | Caffeic acid-3'-glucuronide | 4'-Hydroxycinnamic acid-3'-glucuronide | 0.018 |
| Phenolic acids | Cinnamic acids | Isoferulic acid-3'-glucuronide | 4'-Methoxycinnamic acid-3'-glucuronide | 0.001 |
| Phenolic acids | Cinnamic acids | o-coumaric acid | 2'-Hydroxycinnamic acid | 0.003 |
| Phenolic acids | Hydroxybenzoic acids | Protocatechuic acid-3-sulfate | 4-Hydroxybenzoic acid-3-sulfate | 0.002 |
| Phenolic acids | Hydroxybenzoic acids | 2,3-Dihydroxybenzoic acid | 2,3-Dihydroxybenzoic acid | 0.009 |
| Phenolic acids | Hydroxybenzoic acids | 2,5-Dihydroxybenzoic acid | 2,5-Dihydroxybenzoic acid | 0.047 |
| Phenolic acids | Hydroxybenzoic acids | 2-Hydroxybenzoic acid | 2-Hydroxybenzoic acid | 0.020 |
| Phenolic acids | Hydroxybenzoic acids | Isovanillic acid-3-sulfate | 4-Methoxybenzoic acid-3-sulfate | 0.037 |
| Phenolic acids | Hydroxybenzoic acids | Protocatechuic acid | 3,4-Dihydroxybenzoic acid | 0.007 |
| Phenolic acids | Hydroxybenzoic acids | 2,6-Dihydroxybenzoic acid | 2,6-Dihydroxybenzoic acid | 0.004 |
| Phenolic acids | Hydroxybenzoic acids | 2,4-Dihydroxybenzoic acid | 2,4-Dihydroxybenzoic acid | 0.013 |
| Phenolic acids | Hydroxybenzoic acids | Syringic acid | 4-Hydroxy-3,5-dimethoxybenzoic acid | 0.009 |
| Phenolic acids | Hydroxybenzoic acids | 2-Hydroxy-4-methoxybenzoic acid | 2-Hydroxy-4-methoxybenzoic acid | 0.064 |

**Table S4. Overview of metabolites included for the dietary score DASH**

| **Class** | **Subclass** | **Individual metabolite**  **(Common name)** | **Individual metabolite**  **(Recommended name)** | **Coefficient** |
| --- | --- | --- | --- | --- |
| Flavonoids | Dihydrochalcones | Phloretin | Phloretin | 0.030 |
| Flavonoids | Flavonols | Quercetin-7-glucuronide | Quercetin 7-glucuronide | 0.089 |
| Flavonoids | Flavonols | Quercetin | Quercetin | 0.051 |
| Lignans | Lignans | Enterodiol | Enterodiol | 0.080 |
| Lignans | Lignans | Enterolactone-glucuronide | Enterolactone-glucuronide | 0.017 |
| Lignans | Lignans | Enterolactone-sulfate | Enterolactone-sulfate | 0.033 |
| Other (poly)phenols | Benzene diols and triols | Catechol-1-glucuronide | 2-Hydroxybenzene-1-glucuronide | 0.021 |
| Other (poly)phenols | Tyrosols | Tyrosol | 2-(4-hydroxyphenyl)ethanol | 0.044 |
| Phenolic acids | Cinnamic acids | Cinnamic acid | Cinnamic acid | 0.052 |
| Phenolic acids | Cinnamic acids | Caffeic acid-4'-sulfate | 3'-Hydroxycinnamic acid-4'-sulfate | 0.019 |
| Phenolic acids | Cinnamic acids | Caffeic acid-4'-glucuronide | 3'-Hydroxycinnamic acid-4'-glucuronide | -0.001 |
| Phenolic acids | Cinnamic acids | p-coumaric acid-4'-sulfate | Cinnamic acid-4'-sulfate | 0.052 |
| Phenolic acids | Cinnamic acids | p-coumaric acid-4'-glucuronide | Cinnamic acid-4'-glucuronide | 0.039 |
| Phenolic acids | Cinnamic acids | Caffeic acid-3'-glucuronide | 4'-Hydroxycinnamic acid-3'-glucuronide | 0.030 |
| Phenolic acids | Cinnamic acids | Caffeic acid | 3',4'-Dihydroxycinnamic acid | 0.046 |
| Phenolic acids | Cinnamic acids | Isoferulic acid-3'-glucuronide | 4'-Methoxycinnamic acid-3'-glucuronide | 0.000 |
| Phenolic acids | Cinnamic acids | p-coumaric acid | 4'-Hydroxycinnamic acid | 0.011 |
| Phenolic acids | Cinnamic acids | o-coumaric acid | 2'-Hydroxycinnamic acid | 0.070 |
| Phenolic acids | Hippuric acids | Hippuric acid | Hippuric acid | -0.003 |
| Phenolic acids | Hippuric acids | 2'-Hydroxyhippuric acid | 2'-Hydroxyhippuric acid | 0.042 |
| Phenolic acids | Hydroxybenzoic acids | Protocatechuic acid-4-sulfate | 3-Hydroxybenzoic acid-4-sulfate | 0.034 |
| Phenolic acids | Hydroxybenzoic acids | Protocatechuic acid-3-sulfate | 4-Hydroxybenzoic acid-3-sulfate | 0.010 |
| Phenolic acids | Hydroxybenzoic acids | 2,3-Dihydroxybenzoic acid | 2,3-Dihydroxybenzoic acid | 0.009 |
| Phenolic acids | Hydroxybenzoic acids | 2,5-Dihydroxybenzoic acid | 2,5-Dihydroxybenzoic acid | 0.025 |
| Phenolic acids | Hydroxybenzoic acids | 2-Hydroxybenzoic acid | 2-Hydroxybenzoic acid | 0.077 |
| Phenolic acids | Hydroxybenzoic acids | Isovanillic acid-3-sulfate | 4-Methoxybenzoic acid-3-sulfate | 0.069 |
| Phenolic acids | Hydroxybenzoic acids | Protocatechuic acid | 3,4-Dihydroxybenzoic acid | 0.022 |
| Phenolic acids | Hydroxybenzoic acids | 2,6-Dihydroxybenzoic acid | 2,6-Dihydroxybenzoic acid | 0.050 |
| Phenolic acids | Hydroxybenzoic acids | 2,4-Dihydroxybenzoic acid | 2,4-Dihydroxybenzoic acid | 0.025 |
| Phenolic acids | Hydroxybenzoic acids | 2-Hydroxy-4-methoxybenzoic acid | 2-Hydroxy-4-methoxybenzoic acid | 0.176 |
| Phenolic acids | Phenylacetic acids | 3-(3',5'-Dihydroxyphenyl)propanoic acid | 3-(3',5'-Dihydroxyphenyl)propanoic acid | 0.020 |
| Phenolic acids | Phenylacetic acids | 3-(2',4'-Dihydroxyphenyl)propanoic acid | 3-(2',4'-Dihydroxyphenyl)propanoic acid | 0.008 |
| Phenolic acids | Phenylacetic acids | 2-(4'-Hydroxyphenoxy)propanoic acid | 2-(4'-Hydroxyphenoxy)propanoic acid | 0.033 |
| Phenolic acids | Phenylacetic acids | Dihydrocaffeic acid-3'-sulfate | 3-(4'-Hydroxyphenyl)propanoic acid-3'-sulfate | 0.048 |
| Stilbenes | Stilbenes | Dihydroresveratrol | Dihydroresveratrol | 0.160 |

**Table S5. Overview of metabolites included for the dietary score MIND**

| **Class** | **Subclass** | **Individual metabolite**  **(Common name)** | **Individual metabolite (Recommended name)** | **Coefficient** |
| --- | --- | --- | --- | --- |
| Flavonoids | Dihydrochalcones | Phloretin | Phloretin | 0.024 |
| Flavonoids | Flavonols | Quercetin | Quercetin | 0.051 |
| Lignans | Lignans | Enterodiol | Enterodiol | 0.015 |
| Lignans | Lignans | Enterolactone-glucuronide | Enterolactone-glucuronide | 0.016 |
| Lignans | Lignans | Enterolactone-sulfate | Enterolactone-sulfate | 0.021 |
| Other (poly)phenols | Benzaldehydes | 4-Hydroxybenzaldehyde | 4-Hydroxybenzaldehyde | -0.018 |
| Phenolic acids | Cinnamic acids | Cinnamic acid | Cinnamic acid | 0.030 |
| Phenolic acids | Cinnamic acids | p-coumaric acid-4'-sulfate | Cinnamic acid-4'-sulfate | 0.014 |
| Phenolic acids | Cinnamic acids | p-coumaric acid-4'-glucuronide | Cinnamic acid-4'-glucuronide | 0.014 |
| Phenolic acids | Cinnamic acids | p-coumaric acid | 4'-Hydroxycinnamic acid | 0.014 |
| Phenolic acids | Cinnamic acids | o-coumaric acid | 2'-Hydroxycinnamic acid | -0.005 |
| Phenolic acids | Hippuric acids | alpha-hydroxyhippuric acid | alpha-hydroxyhippuric acid | -0.011 |
| Phenolic acids | Hydroxybenzoic acids | Protocatechuic acid-4-sulfate | 3-Hydroxybenzoic acid-4-sulfate | 0.011 |
| Phenolic acids | Hydroxybenzoic acids | Isovanillic acid-3-sulfate | 4-Methoxybenzoic acid-3-sulfate | 0.018 |
| Phenolic acids | Phenylacetic acids | 3-(3'-Hydroxyphenyl)propanoic acid | 3-(3'-Hydroxyphenyl)propanoic acid | 0.009 |

**Table S6. Overview of metabolites included for the dietary score hPDI**

| **Class** | **Subclass** | **Individual metabolite**  **(Common name)** | **Individual metabolite**  **(Recommended name)** | **Coefficient** |
| --- | --- | --- | --- | --- |
| Flavonoids | Flavonols | Quercetin-3-glucuronide | Quercetin 3-glucuronide | 0.051 |
| Flavonoids | Flavonols | Quercetin-7-glucuronide | Quercetin 7-glucuronide | 0.184 |
| Flavonoids | Flavonols | Quercetin | Quercetin | 0.100 |
| Lignans | Lignans | Enterodiol | Enterodiol | 0.139 |
| Lignans | Lignans | Enterolactone-glucuronide | Enterolactone-glucuronide | 0.068 |
| Lignans | Lignans | Enterolactone-sulfate | Enterolactone-sulfate | 0.068 |
| Other (poly)phenols | Benzene diols and triols | Catechol-1-glucuronide | 2-Hydroxybenzene-1-glucuronide | 0.051 |
| Other (poly)phenols | Hydroxycoumarins | Urolithin B | 3-Hydroxy-urolithin | 0.011 |
| Other (poly)phenols | Tyrosols | Hydroxytyrosol-4'-sulfate | 3'-Hydroxyphenylethanol-4'-sulfate | 0.086 |
| Other (poly)phenols | Tyrosols | Tyrosol | 2-(4-hydroxyphenyl)ethanol | 0.018 |
| Phenolic acids | Cinnamic acids | Cinnamic acid | Cinnamic acid | 0.144 |
| Phenolic acids | Cinnamic acids | Caffeic acid-4'-glucuronide | 3'-Hydroxycinnamic acid-4'-glucuronide | -0.017 |
| Phenolic acids | Cinnamic acids | p-coumaric acid-4'-sulfate | Cinnamic acid-4'-sulfate | 0.041 |
| Phenolic acids | Cinnamic acids | p-coumaric acid-4'-glucuronide | Cinnamic acid-4'-glucuronide | 0.064 |
| Phenolic acids | Cinnamic acids | Caffeic acid-3'-glucuronide | 4'-Hydroxycinnamic acid-3'-glucuronide | 0.038 |
| Phenolic acids | Cinnamic acids | Caffeic acid | 3',4'-Dihydroxycinnamic acid | 0.030 |
| Phenolic acids | Cinnamic acids | Isoferulic acid-3'-glucuronide | 4'-Methoxycinnamic acid-3'-glucuronide | 0.033 |
| Phenolic acids | Cinnamic acids | p-coumaric acid | 4'-Hydroxycinnamic acid | 0.001 |
| Phenolic acids | Cinnamic acids | o-coumaric acid | 2'-Hydroxycinnamic acid | 0.069 |
| Phenolic acids | Hippuric acids | 2'-Hydroxyhippuric acid | 2'-Hydroxyhippuric acid | 0.058 |
| Phenolic acids | Hydroxybenzoic acids | 2,3-Dihydroxybenzoic acid | 2,3-Dihydroxybenzoic acid | 0.027 |
| Phenolic acids | Hydroxybenzoic acids | 2,5-Dihydroxybenzoic acid | 2,5-Dihydroxybenzoic acid | 0.100 |
| Phenolic acids | Hydroxybenzoic acids | 2-Hydroxybenzoic acid | 2-Hydroxybenzoic acid | 0.074 |
| Phenolic acids | Hydroxybenzoic acids | Isovanillic acid-3-sulfate | 4-Methoxybenzoic acid-3-sulfate | 0.066 |
| Phenolic acids | Hydroxybenzoic acids | Protocatechuic acid | 3,4-Dihydroxybenzoic acid | 0.036 |
| Phenolic acids | Hydroxybenzoic acids | 2,6-Dihydroxybenzoic acid | 2,6-Dihydroxybenzoic acid | 0.017 |
| Phenolic acids | Hydroxybenzoic acids | 3-Hydroxybenzoic acid | 3-Hydroxybenzoic acid | 0.054 |
| Phenolic acids | Hydroxybenzoic acids | 2,4-Dihydroxybenzoic acid | 2,4-Dihydroxybenzoic acid | 0.014 |
| Phenolic acids | Hydroxybenzoic acids | Benzoic acid | Benzoic acid | 0.080 |
| Phenolic acids | Hydroxybenzoic acids | 2-Hydroxy-4-methoxybenzoic acid | 2-Hydroxy-4-methoxybenzoic acid | 0.216 |
| Phenolic acids | Phenylacetic acids | 2-(4'-Hydroxyphenoxy)propanoic acid | 2-(4'-Hydroxyphenoxy)propanoic acid | 0.041 |
| Phenolic acids | Phenylacetic acids | Dihydrocaffeic acid-3'-sulfate | 3-(4'-Hydroxyphenyl)propanoic acid-3'-sulfate | 0.056 |
| Stilbenes | Stilbenes | Dihydroresveratrol | Dihydroresveratrol | 0.152 |

**Table S7. Overview of metabolites included for the dietary score uPDI**

| **Class** | **Subclass** | **Individual metabolite**  **(Common name)** | **Individual metabolite**  **(Recommended name)** | **Coefficient** |
| --- | --- | --- | --- | --- |
| Methylxanthines | Methylxanthines | Caffeine | Caffeine | -0.043 |
| Methylxanthines | Methylxanthines | Theophylline | Theophylline | -0.083 |
| Methylxanthines | Methylxanthines | Paraxanthine | Paraxanthine | -0.052 |
| Flavonoids | Dihydrochalcones | Phloretin | Phloretin | -0.135 |
| Flavonoids | Flavonols | Quercetin-7-glucuronide | Quercetin 7-glucuronide | -0.097 |
| Lignans | Lignans | Enterodiol | Enterodiol | -0.376 |
| Lignans | Lignans | Enterolactone-glucuronide | Enterolactone-glucuronide | -0.032 |
| Lignans | Lignans | Enterolactone-sulfate | Enterolactone-sulfate | -0.029 |
| Other (poly)phenols | Benzene diols and triols | Catechol-1-glucuronide | 2-Hydroxybenzene-1-glucuronide | 0.057 |
| Other (poly)phenols | Tyrosols | Tyrosol | 2-(4-hydroxyphenyl)ethanol | -0.044 |
| Phenolic acids | Cinnamic acids | Cinnamic acid | Cinnamic acid | 0.034 |
| Phenolic acids | Cinnamic acids | Caffeic acid-4'-sulfate | 3'-Hydroxycinnamic acid-4'-sulfate | -0.016 |
| Phenolic acids | Cinnamic acids | Caffeic acid-4'-glucuronide | 3'-Hydroxycinnamic acid-4'-glucuronide | -0.101 |
| Phenolic acids | Cinnamic acids | p-coumaric acid-4'-sulfate | Cinnamic acid-4'-sulfate | -0.059 |
| Phenolic acids | Cinnamic acids | Caffeic acid-3'-glucuronide | 4'-Hydroxycinnamic acid-3'-glucuronide | 0.022 |
| Phenolic acids | Cinnamic acids | Caffeic acid | 3',4'-Dihydroxycinnamic acid | -0.138 |
| Phenolic acids | Cinnamic acids | Chlorogenic acid | 5-O-Caffeoylquinic acid | 0.003 |
| Phenolic acids | Cinnamic acids | Cryptochlorogenic acid | 4-O-Caffeoylquinic acid | -0.038 |
| Phenolic acids | Cinnamic acids | Isoferulic acid-3'-glucuronide | 4'-Methoxycinnamic acid-3'-glucuronide | 0.038 |
| Phenolic acids | Cinnamic acids | 4-O-Feruloylquinic acid | 4-O-Feruloylquinic acid | -0.011 |
| Phenolic acids | Cinnamic acids | o-coumaric acid | 2'-Hydroxycinnamic acid | -0.091 |
| Phenolic acids | Hippuric acids | Hippuric acid | Hippuric acid | -0.040 |
| Phenolic acids | Hippuric acids | 2'-Hydroxyhippuric acid | 2'-Hydroxyhippuric acid | -0.086 |
| Phenolic acids | Hydroxybenzoic acids | Protocatechuic acid-3-sulfate | 4-Hydroxybenzoic acid-3-sulfate | -0.009 |
| Phenolic acids | Hydroxybenzoic acids | 2-Hydroxybenzoic acid | 2-Hydroxybenzoic acid | -0.182 |
| Phenolic acids | Hydroxybenzoic acids | Isovanillic acid-3-sulfate | 4-Methoxybenzoic acid-3-sulfate | -0.116 |
| Phenolic acids | Hydroxybenzoic acids | Benzoic acid | Benzoic acid | -0.105 |
| Phenolic acids | Hydroxybenzoic acids | 2-Hydroxy-4-methoxybenzoic acid | 2-Hydroxy-4-methoxybenzoic acid | -0.180 |
| Phenolic acids | Phenylacetic acids | 2-(4'-Hydroxyphenoxy)propanoic acid | 2-(4'-Hydroxyphenoxy)propanoic acid | 0.014 |
| Phenolic acids | Phenylacetic acids | 3-(2',3'-Dihydroxyphenyl)propanoic acid | 3-(2',3'-Dihydroxyphenyl)propanoic acid | -0.057 |
| Phenolic acids | Phenylacetic acids | Dihydrocaffeic acid-3'-sulfate | 3-(4'-Hydroxyphenyl)propanoic acid-3'-sulfate | -0.078 |
| Stilbenes | Stilbenes | Dihydroresveratrol | Dihydroresveratrol | -0.279 |
| Stilbenes | Stilbenes | cis-Resveratrol-4'-glucuronide | cis-Resveratrol-4'-glucuronide | -0.089 |

**Table S8. Comparison of correlation between metabolic signature and significant individual metabolites**

| **Class** | **Subclass** | **Individual metabolite  (Common name)** | **Individual metabolite  (Recommended name)** | **A-MED** | **O-MED** | **DASH** | **MIND** | **hPDI** | **uPDI** |
| --- | --- | --- | --- | --- | --- | --- | --- | --- | --- |
| **Metabolic signature** | | | | **0.359** | **0.254** | **0.374** | **0.342** | **0.353** | **0.367** |
| **Significantly associated metabolites** | | | | | | | | | |
| Methylxanthines | Methylxanthines | Caffeine | Caffeine |  |  |  |  |  | -0.242 |
| Methylxanthines | Methylxanthines | Theophylline | Theophylline |  |  |  |  |  | -0.228 |
| Methylxanthines | Methylxanthines | Paraxanthine | Paraxanthine |  |  |  |  |  | -0.196 |
| Flavonoids | Dihydrochalcones | Phloretin | Phloretin | 0.145 |  | 0.186 | 0.140 |  | -0.170 |
| Flavonoids | Flavanols | (-)-Epicatechin | (-)-Epicatechin | 0.148 | 0.181 |  |  |  |  |
| Flavonoids | Flavanones | Naringenin-4'-glucuronide | Naringenin-4'-glucuronide | 0.154 |  |  |  |  |  |
| Flavonoids | Flavonols | Quercetin-3-glucuronide | Quercetin 3-glucuronide | 0.172 |  |  |  | 0.161 |  |
| Flavonoids | Flavonols | Quercetin-7-glucuronide | Quercetin 7-glucuronide | 0.190 |  | 0.175 |  | 0.172 | -0.162 |
| Flavonoids | Flavonols | Quercetin | Quercetin | 0.183 | 0.146 | 0.169 | 0.179 | 0.190 |  |
| Lignans | Lignans | Enterolactone-glucuronide | Enterolactone-glucuronide | 0.208 | 0.144 | 0.234 | 0.146 | 0.232 | -0.166 |
| Lignans | Lignans | Enterolactone-sulfate | Enterolactone-sulfate | 0.213 | 0.150 | 0.273 | 0.171 | 0.264 | -0.148 |
| Lignans | Lignans | Enterodiol | Enterodiol |  |  | 0.184 | 0.136 | 0.213 | -0.271 |
| Other (poly)phenols | Benzaldehydes | 4-Hydroxybenzaldehyde | 4-Hydroxybenzaldehyde | 0.190 | 0.133 |  | 0.155 |  |  |
| Other (poly)phenols | Benzene diols and triols | Catechol-1-glucuronide | 2-Hydroxybenzene-1-glucuronide | 0.188 |  | 0.208 |  | 0.219 | -0.184 |
| Other (poly)phenols | Hydroxycoumarins | Urolithin B | 3-Hydroxy-urolithin |  |  |  |  | 0.156 |  |
| Other (poly)phenols | Tyrosols | Tyrosol | 2-(4-hydroxyphenyl)ethanol | 0.143 | 0.149 | 0.186 |  | 0.152 | -0.204 |
| Other (poly)phenols | Tyrosols | Hydroxytyrosol-4'-sulfate | 3'-Hydroxyphenylethanol-4'-sulfate |  |  |  |  | 0.153 |  |
| Phenolic acids | Cinnamic acids | Cinnamic acid | Cinnamic acid | 0.189 | 0.160 | 0.180 | 0.152 | 0.216 | -0.154 |
| Phenolic acids | Cinnamic acids | Caffeic acid-4'-sulfate | 3'-Hydroxycinnamic acid-4'-sulfate | 0.147 |  | 0.168 |  |  | -0.144 |
| Phenolic acids | Cinnamic acids | p-coumaric acid-4'-sulfate | Cinnamic acid-4'-sulfate | 0.260 | 0.186 | 0.259 | 0.207 | 0.202 | -0.172 |
| Phenolic acids | Cinnamic acids | p-coumaric acid-4'-glucuronide | Cinnamic acid-4'-glucuronide | 0.275 | 0.175 | 0.235 | 0.187 | 0.189 |  |
| Phenolic acids | Cinnamic acids | Caffeic acid-3'-glucuronide | 4'-Hydroxycinnamic acid-3'-glucuronide | 0.184 | 0.144 | 0.194 |  | 0.196 | -0.157 |
| Phenolic acids | Cinnamic acids | Caffeic acid | 3',4'-Dihydroxycinnamic acid | 0.204 |  | 0.240 |  | 0.184 | -0.282 |
| Phenolic acids | Cinnamic acids | Cryptochlorogenic acid | 4-O-Caffeoylquinic acid | 0.147 |  |  |  |  | -0.201 |
| Phenolic acids | Cinnamic acids | Isoferulic acid-3'-glucuronide | 4'-Methoxycinnamic acid-3'-glucuronide | 0.208 | 0.143 | 0.157 |  | 0.185 | -0.151 |
| Phenolic acids | Cinnamic acids | Sinapic acid | 4'-Hydroxy-3',5'-dimethoxycinnamic acid | 0.152 |  |  |  |  |  |
| Phenolic acids | Cinnamic acids | o-coumaric acid | 2'-Hydroxycinnamic acid | 0.194 | 0.172 | 0.262 | 0.183 | 0.253 | -0.164 |
| Phenolic acids | Cinnamic acids | Caffeic acid-4'-glucuronide | 3'-Hydroxycinnamic acid-4'-glucuronide |  |  | 0.159 |  | 0.147 | -0.218 |
| Phenolic acids | Cinnamic acids | p-coumaric acid | 4'-Hydroxycinnamic acid |  |  | 0.165 | 0.142 | 0.144 |  |
| Phenolic acids | Cinnamic acids | Chlorogenic acid | 5-O-Caffeoylquinic acid |  |  |  |  |  | -0.145 |
| Phenolic acids | Cinnamic acids | 4-O-Feruloylquinic acid | 4-O-Feruloylquinic acid |  |  |  |  |  | -0.161 |
| Phenolic acids | Hippuric acids | Hippuric acid | Hippuric acid | 0.150 |  | 0.174 |  |  | -0.176 |
| Phenolic acids | Hippuric acids | 2'-Hydroxyhippuric acid | 2'-Hydroxyhippuric acid |  |  | 0.194 |  | 0.187 | -0.163 |
| Phenolic acids | Hippuric acids | alpha-hydroxyhippuric acid | alpha-hydroxyhippuric acid |  |  |  | -0.179 |  |  |
| Phenolic acids | Hydroxybenzoic acids | Protocatechuic acid-4-sulfate | 3-Hydroxybenzoic acid-4-sulfate | 0.153 |  | 0.170 | 0.136 |  |  |
| Phenolic acids | Hydroxybenzoic acids | Protocatechuic acid-3-sulfate | 4-Hydroxybenzoic acid-3-sulfate | 0.173 | 0.146 | 0.159 |  |  | -0.142 |
| Phenolic acids | Hydroxybenzoic acids | 2,3-Dihydroxybenzoic acid | 2,3-Dihydroxybenzoic acid | 0.229 | 0.165 | 0.168 |  | 0.182 |  |
| Phenolic acids | Hydroxybenzoic acids | 2,5-Dihydroxybenzoic acid | 2,5-Dihydroxybenzoic acid | 0.231 | 0.207 | 0.202 |  | 0.222 |  |
| Phenolic acids | Hydroxybenzoic acids | 2-Hydroxybenzoic acid | 2-Hydroxybenzoic acid | 0.191 | 0.174 | 0.231 |  | 0.205 | -0.259 |
| Phenolic acids | Hydroxybenzoic acids | Isovanillic acid-3-sulfate | 4-Methoxybenzoic acid-3-sulfate | 0.254 | 0.168 | 0.286 | 0.145 | 0.240 | -0.215 |
| Phenolic acids | Hydroxybenzoic acids | Protocatechuic acid | 3,4-Dihydroxybenzoic acid | 0.193 | 0.178 | 0.150 |  | 0.142 |  |
| Phenolic acids | Hydroxybenzoic acids | 2,6-Dihydroxybenzoic acid | 2,6-Dihydroxybenzoic acid | 0.220 | 0.152 | 0.294 |  | 0.254 |  |
| Phenolic acids | Hydroxybenzoic acids | 2,4-Dihydroxybenzoic acid | 2,4-Dihydroxybenzoic acid | 0.201 | 0.146 | 0.246 |  | 0.225 |  |
| Phenolic acids | Hydroxybenzoic acids | Vanillic acid | 4-Hydroxy-3-methoxybenzoic acid | 0.143 |  |  |  |  |  |
| Phenolic acids | Hydroxybenzoic acids | Syringic acid | 4-Hydroxy-3,5-dimethoxybenzoic acid | 0.182 | 0.145 |  |  |  |  |
| Phenolic acids | Hydroxybenzoic acids | 2-Hydroxy-4-methoxybenzoic acid | 2-Hydroxy-4-methoxybenzoic acid | 0.229 | 0.171 | 0.241 |  | 0.165 | -0.262 |
| Phenolic acids | Hydroxybenzoic acids | 3-Hydroxybenzoic acid | 3-Hydroxybenzoic acid |  |  |  |  | 0.145 |  |
| Phenolic acids | Hydroxybenzoic acids | Benzoic acid | Benzoic acid |  |  |  |  | 0.164 | -0.178 |
| Phenolic acids | Phenylacetic acids | 3-(3',5'-Dihydroxyphenyl)propanoic acid | 3-(3',5'-Dihydroxyphenyl)propanoic acid | 0.157 |  | 0.160 |  |  |  |
| Phenolic acids | Phenylacetic acids | 3-(2',4'-Dihydroxyphenyl)propanoic acid | 3-(2',4'-Dihydroxyphenyl)propanoic acid | 0.153 |  | 0.151 |  |  |  |
| Phenolic acids | Phenylacetic acids | Dihydrocaffeic acid | 3-(3',4'-Dihydroxyphenyl)propanoic acid | 0.191 |  |  |  |  |  |
| Phenolic acids | Phenylacetic acids | 2-(4'-Hydroxyphenoxy)propanoic acid | 2-(4'-Hydroxyphenoxy)propanoic acid | 0.192 |  | 0.191 |  | 0.161 | -0.167 |
| Phenolic acids | Phenylacetic acids | 3-(2',3'-Dihydroxyphenyl)propanoic acid | 3-(2',3'-Dihydroxyphenyl)propanoic acid | 0.192 |  |  |  |  | -0.183 |
| Phenolic acids | Phenylacetic acids | Dihydrocaffeic acid-3'-sulfate | 3-(4'-Hydroxyphenyl)propanoic acid-3'-sulfate | 0.196 |  | 0.161 |  | 0.159 | -0.180 |
| Phenolic acids | Phenylacetic acids | 3-(3'-Hydroxyphenyl)propanoic acid | 3-(3'-Hydroxyphenyl)propanoic acid |  |  |  | 0.149 |  |  |
| Stilbenes | Stilbenes | Dihydroresveratrol | Dihydroresveratrol | 0.189 |  | 0.227 |  | 0.151 | -0.243 |
| Stilbenes | Stilbenes | trans-Resveratrol-3-glucuronide | trans-Resveratrol-3-glucuronide | 0.167 |  |  |  |  |  |
| Stilbenes | Stilbenes | cis-Resveratrol-4'-glucuronide | cis-Resveratrol-4'-glucuronide |  |  |  |  |  | -0.149 |

**Table S9. Cross-validation between dietary pattern and corresponding metabolic signature in each dataset**

|  | **A-MED** | **O-MED** | **DASH** | **MIND** | **hPDI** | **uPDI** |
| --- | --- | --- | --- | --- | --- | --- |
| **Derivation dataset** |  |  |  |  |  |  |
| Correctly classified (%) ^1^ | 77.5 | 72.5 | 77.1 | 75.2 | 75.2 | 72.9 |
| Opposite quartile (%) | 4.6 | 7.3 | 5.5 | 4.6 | 8.3 | 3.7 |
| **Validation dataset** |  |  |  |  |  |  |
| POLYNTAKE cohort with plasma sample (n = 195) | |  |  |  |  |  |
| Correctly classified (%) ^1^ | 65.6 | 73.3 | 70.8 | 69.7 | 70.3 | 65.1 |
| Opposite quartile (%) | 7.2 | 10.8 | 10.8 | 12.8 | 6.2 | 8.7 |
| TwinsUK cohort with spot urine sample (n = 198) | |  |  |  |  |  |
| Correctly classified (%) ^1^ | 68.2 | 72.3 | 69.2 | 66.2 | 75.9 | 70.8 |
| Opposite quartile (%) | 11.0 | 12.0 | 11.0 | 9.0 | 9.0 | 7.0 |
| ABP study with 24h urine sample (n = 88) | |  |  |  |  |  |
| Correctly classified (%) ^1^ | 78.4 | 81.8 | 76.1 | 73.9 | 76.1 | 75.0 |
| Opposite quartile (%) | 4.5 | 8.0 | 6.8 | 4.5 | 9.1 | 5.7 |

1. Corrected classified including same quartiles andadjacent quartiles. DASH, Dietary Approaches to Stop Hypertension; MIND, Mediterranean-DASH Intervention for Neurodegenerative Delay; O-MED, Original Mediterranean Score; A-MED, Amended Mediterranean Score; hPDI, healthy Plant-based Diet Index; uPDI, unhealthy Plant-based Diet Index.


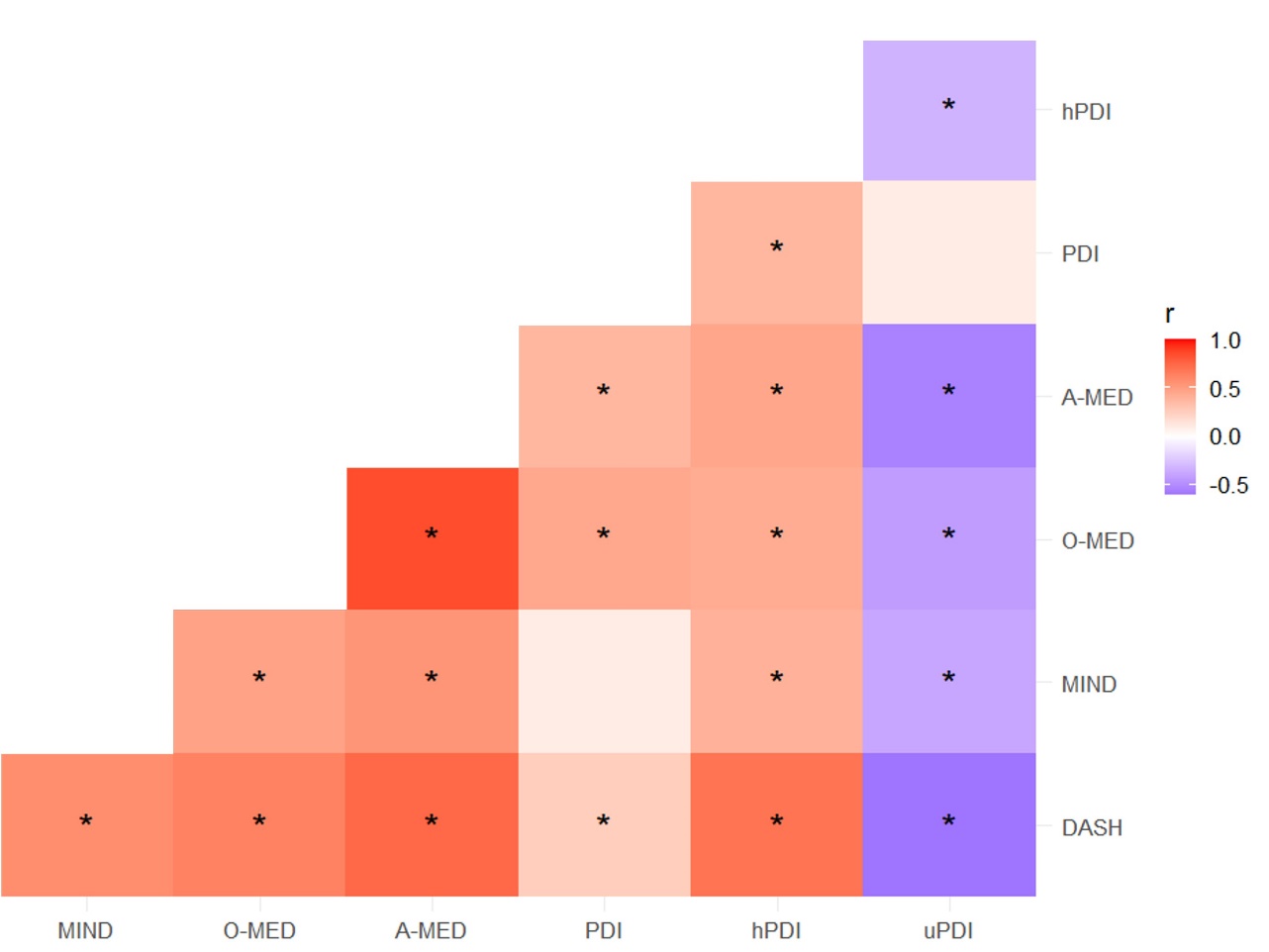
**Figure S1. Correlation among plant-rich dietary scores.**

The colour scale indicates the Spearman correlation coefficient between dietary scores. Red and blue illustrated respectively positive and negative correlations and colour intensity represented the degree of the coefficient. The asterisks showed significance (*FDR-adjusted, *p* < 0.05), DASH, Dietary Approaches to Stop Hypertension; O-MED, Original Mediterranean Score; A-MED, Amended Mediterranean Score; MIND, Mediterranean-DASH Intervention for Neurodegenerative Delay; PDI, Plant-based Diet Index; hPDI, healthy Plant-based Diet Index; uPDI, unhealthy Plant-based Diet Index.


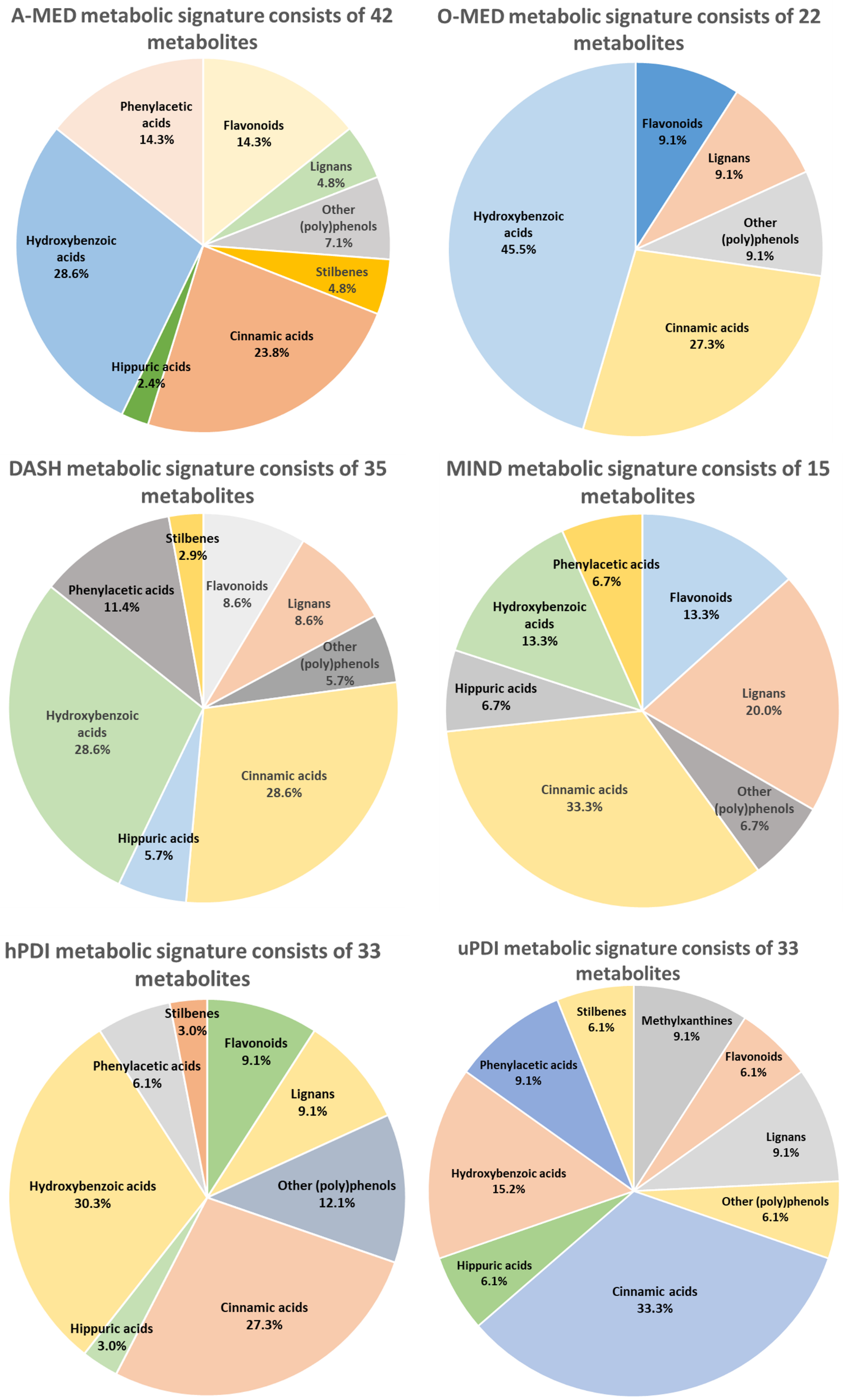


**Figure S2. The percentage of the metabolites in each metabolic signature from derivation set in the POLYNTAKE cohort with 24h urine sample (n = 218).**

DASH, Dietary Approaches to Stop Hypertension; O-MED, Original Mediterranean Score; A-MED, Amended Mediterranean Score; MIND, Mediterranean-DASH Intervention for Neurodegenerative Delay; hPDI, healthy Plant-based Diet Index; uPDI, unhealthy Plant-based Diet Index.

**
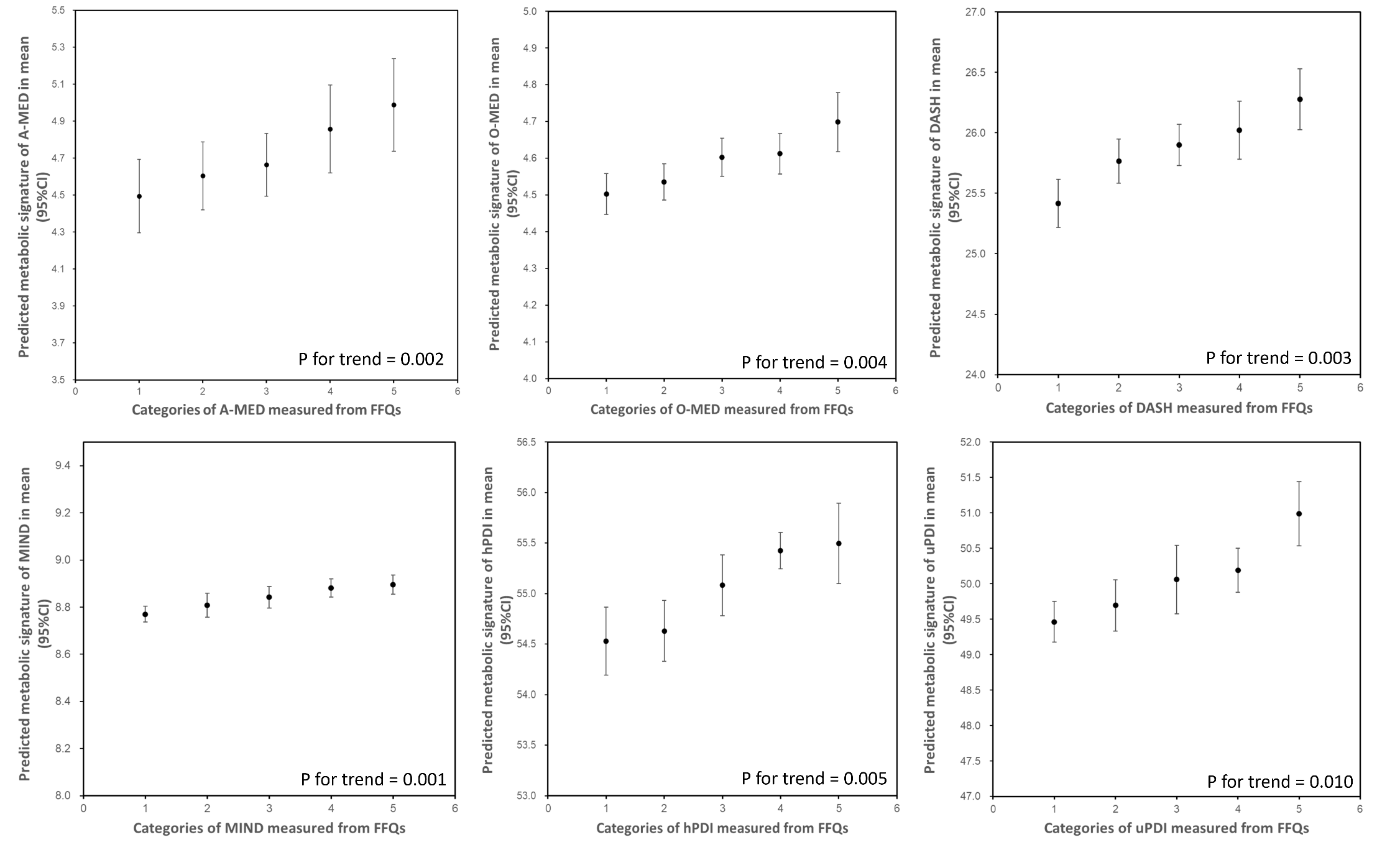
Figure S3. The correlations between metabolic signatures and plant-rich dietary patterns from derivation set in the POLYNTAKE cohort with 24h urine sample (n = 218).**

A. The means and 95% CI of dietary pattern (A-MED) in quintiles of the derived metabolic signature (42 metabolites). B. the means and 95% CI of dietary pattern (O-MED) in quintiles of the derived metabolic signature (22 metabolites). C. The means and 95% CI of dietary pattern (DASH) in quintiles of the derived metabolic signature (35 metabolites). D. The means and 95% CI of dietary pattern (MIND) in quintiles of the derived metabolic signature (15 metabolites). E. The means and 95% CI of dietary pattern (hPDI) in quintiles of the derived metabolic signature (33 metabolites). F. The means and 95% CI of dietary pattern (uPDI) in quintiles of the derived metabolic signature (33 metabolites). DASH, Dietary Approaches to Stop Hypertension; O-MED, Original Mediterranean Score; A-MED, Amended Mediterranean Score; MIND, Mediterranean-DASH Intervention for Neurodegenerative Delay; hPDI, healthy Plant-based Diet Index; uPDI, unhealthy Plant-based Diet Index. P for trend < 0.05


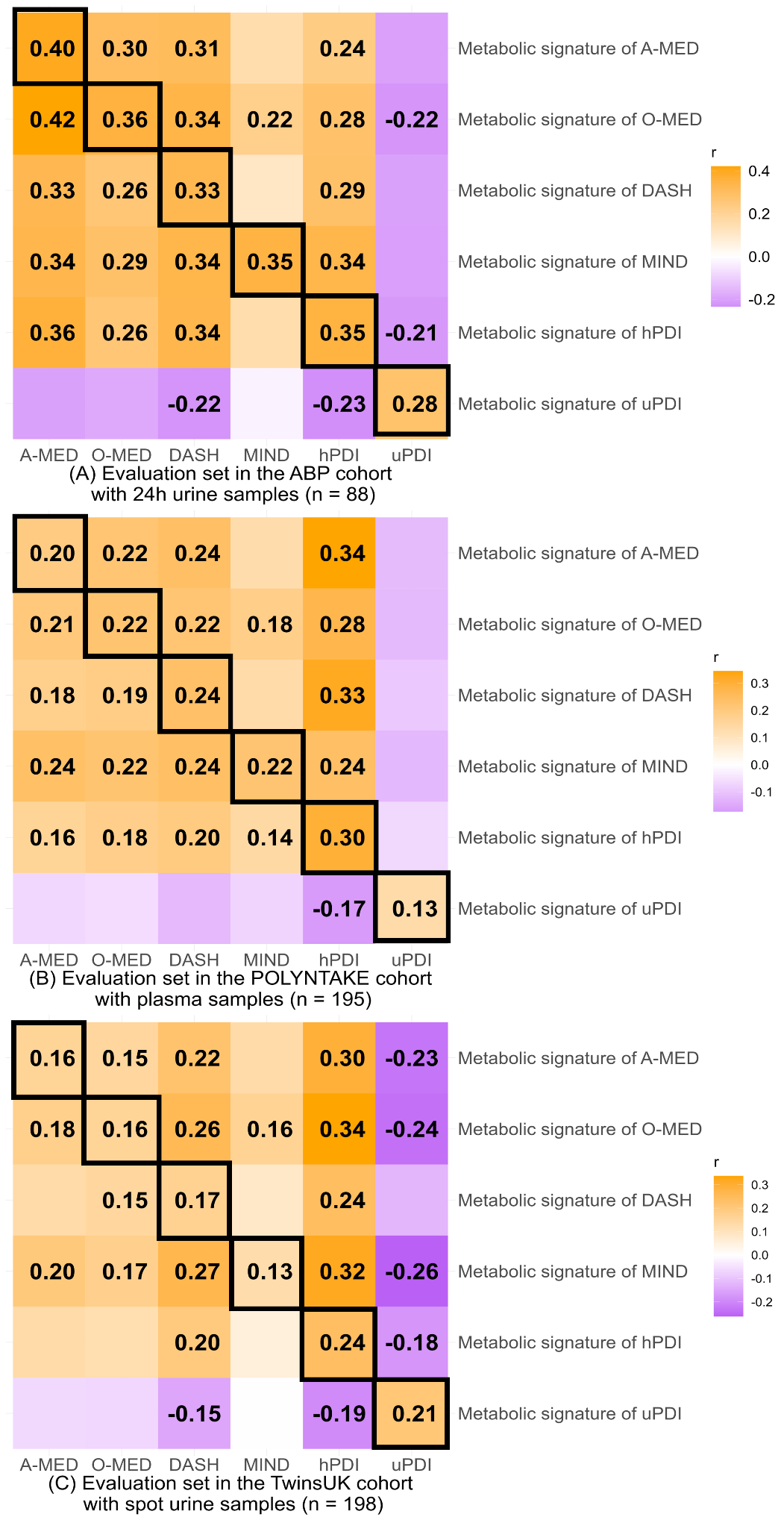


**Figure S4. The correlations matrix for plant-rich dietary patterns and metabolic signatures from validation dataset in the ABP study with 24h urine sample (n = 88), POLYNTAKE cohort with plasma sample (n = 195), and TwinsUK cohort with spot urine sample (n = 198).**

The dietary scores including A-MED, O-MED, DASH, MIND, hPDI, and uPDI measured by FFQs. The metabolic signatures derived based on the selected metabolites that significantly associated with each plant-rich dietary score, respectively. The colour scale indicated the Spearman correlation coefficient between plant-rich dietary patterns and metabolic signature. Red and blue illustrated positive and negative correlations and colour intensity represented the degree of the coefficient. The correlation with significance were listed the coefficient (FDR-adjusted, *p* < 0.05). The signature of uPDI in the POLYNTAKE cohort with plasma sample (FDR-adjusted, *p* = 0.06), and MIND signature in the TwinsUK cohort (FDR-adjusted, *p* = 0.05, 0.06) were listed as well for suggestive significant correlation. DASH, Dietary Approaches to Stop Hypertension; MIND, Mediterranean-DASH Intervention for Neurodegenerative Delay; O-MED, Original Mediterranean Score; A-MED, Amended Mediterranean Score; hPDI, healthy Plant-based Diet Index; uPDI, unhealthy Plant-based Diet Index.


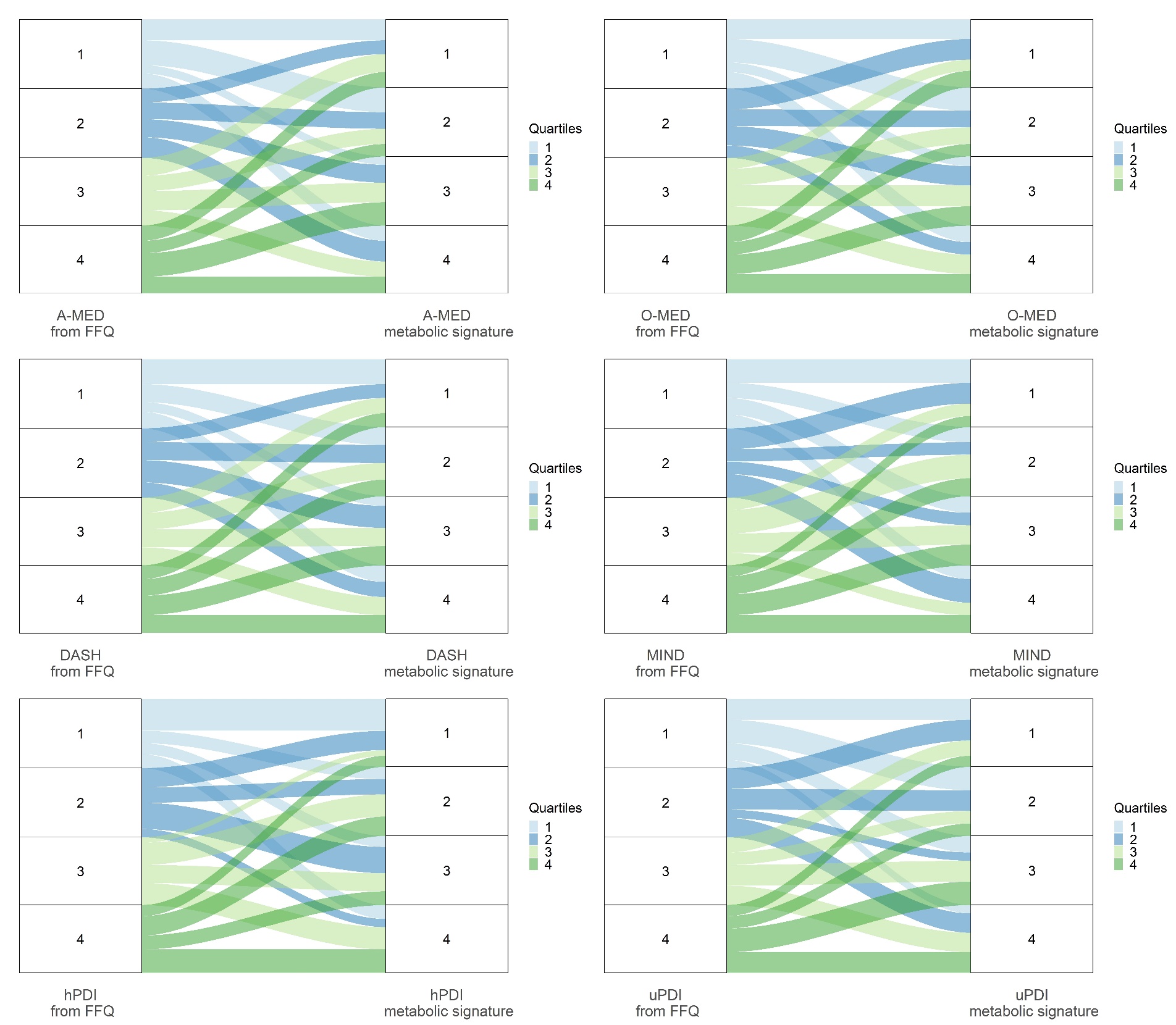


**Figure S5. Agreements between plant-rich dietary patterns and the metabolic signature in ranking participants into quartiles: the TwinsUK Study (spot urine, n = 198).**

DASH, Dietary Approaches to Stop Hypertension; MIND, Mediterranean-DASH Intervention for Neurodegenerative Delay; O-MED, Original Mediterranean Score; A-MED, Amended Mediterranean Score; hPDI, Healthy Plant-based Diet Index; uPDI, unhealthy Plant-based Diet Index.

**
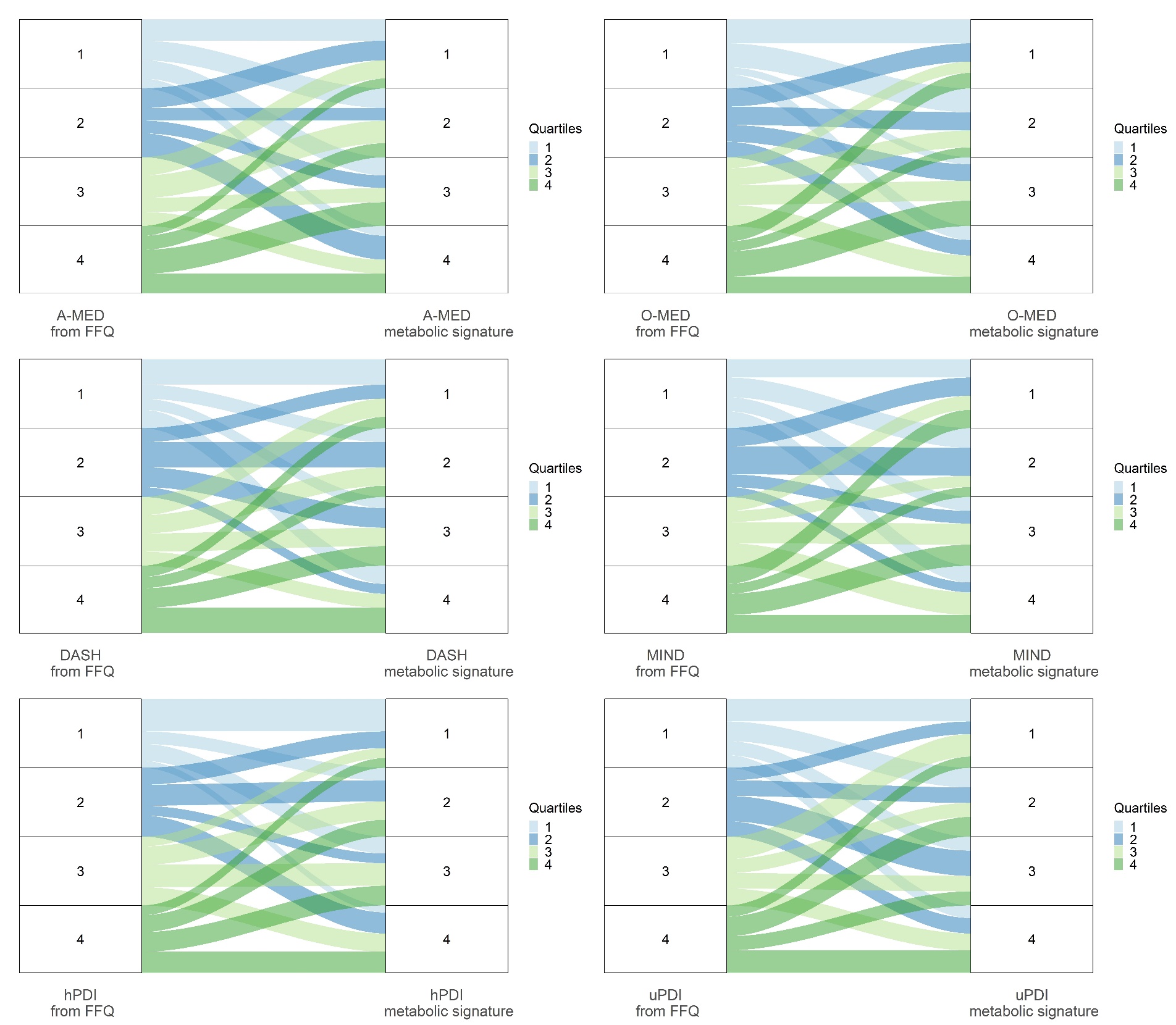
**

**Figure S6. Agreements between plant-rich dietary patterns and the metabolic signature in ranking participants into quartiles: the POLYNTAKE Study (plasma, n = 195).**

DASH, Dietary Approaches to Stop Hypertension; MIND, Mediterranean-DASH Intervention for Neurodegenerative Delay; O-MED, Original Mediterranean Score; A-MED, Amended Mediterranean Score; hPDI, healthy Plant-based Diet Index; uPDI, unhealthy Plant-based Diet Index.


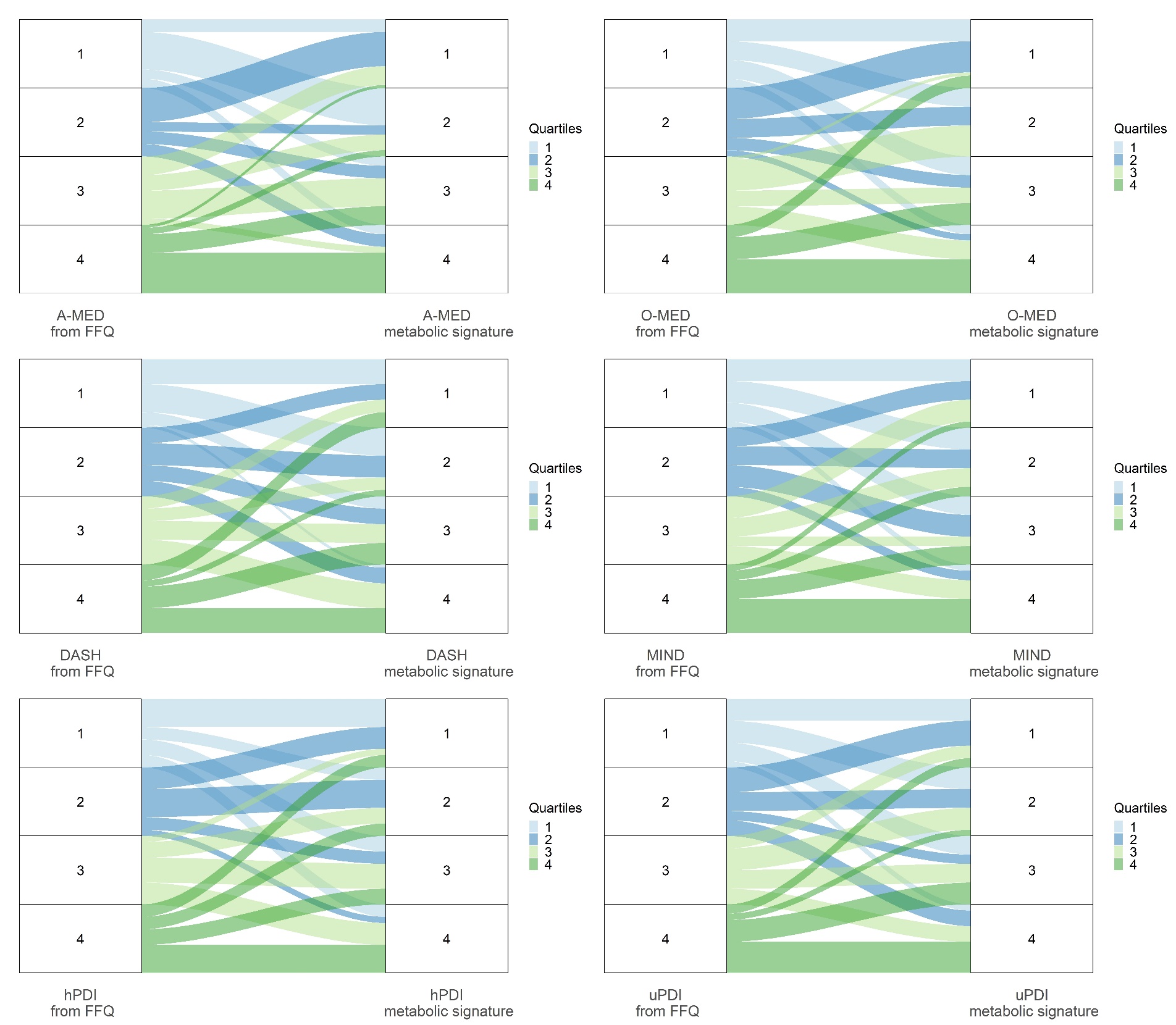


**Figure S7. Agreements between plant-rich dietary patterns and the metabolic signature in ranking participants into quartiles: the ABP Study (24h urine, n = 88).**

DASH, Dietary Approaches to Stop Hypertension; MIND, Mediterranean-DASH Intervention for Neurodegenerative Delay; O-MED, Original Mediterranean Score; A-MED, Amended Mediterranean Score; hPDI, healthy Plant-based Diet Index; uPDI, unhealthy Plant-based Diet Index.
